# Enhanced molecular first hyperpolarizabilities with Reichardt’s type of zwitterions: a computational study on roles of various monocyclic aromatic bridges

**DOI:** 10.1007/s00894-024-06055-3

**Published:** 2024-07-26

**Authors:** Divya Pant, Sanyasi Sitha

**Affiliations:** Department of Chemical Sciences, Auckland Park Kingsway (APK) Campus, University of Johannesburg, PO Box 524, Auckland Park, 2006, Johannesburg, South Africa

**Keywords:** Reichardt’s zwitterions, Dipole moment, Polarizability, Hyperpolarizability, Aromatic bridges

## Abstract

**Context:**

This work reports structure–property correlations of 27 zwitterions Reichardt’s types of zwitterions. Focuses are twofold, to see the (1) impacts of metamerism with Reichardt’s vs Brooker’s types of zwitterions and (2) impacts of monocyclic aromatic rings as bridges. All the molecules considered here have pyridinium (common acceptor: A) and *p*-phenylene-dicyanomethanide (common donor: D). Fundamental molecular properties like dipole moments (*μ*), polarizabilities (*α*), hyperpolarizabilities (*β*), and adiabatic absorptions were computed only for the Reichardt types and compared with the literature reported respective Brooker’s types of zwitterions. As an impact of metamerism, in general 2–3 times enhanced hyperpolarizabilities (*β*) were observed for Reichardt’s compared to Brooker’s types. Exceptions were observed with some triazine bridges and furan bridge, where Brooker’s types were found to be more efficient. As impacts of aromatic bridges, in general, 6–sevenfold enhanced *β* compared to well-known traditional bridges and enhanced *β* were observed compared to D-A directly connected zwitterion (benzene bridge: sixfold enhanced *β*). Current findings show that the aromatic bridge control with Reichardt’s types of zwitterions is more efficient and thus may be employed as an effective strategy for the designing of functional molecular chromophores for various other fundamental areas.

**Methods:**

All computations were performed with Gaussian 09. Geometry optimizations and computations of fundamental properties were carried out with HF, B3LYP, CAM-B3LYP, and ωB97xD methodologies, with 6-31G(d,p) and aug-cc-pVDZ basis sets. For adiabatic excitations, computations were carried out using TDDFT and TDHF approaches. For the computations of the response properties (like the nonlinear optical responses), CPHF approach was used.

**Supplementary information:**

The online version contains supplementary material available at 10.1007/s00894-024-06055-3.

## Introduction

**Scheme 1:**
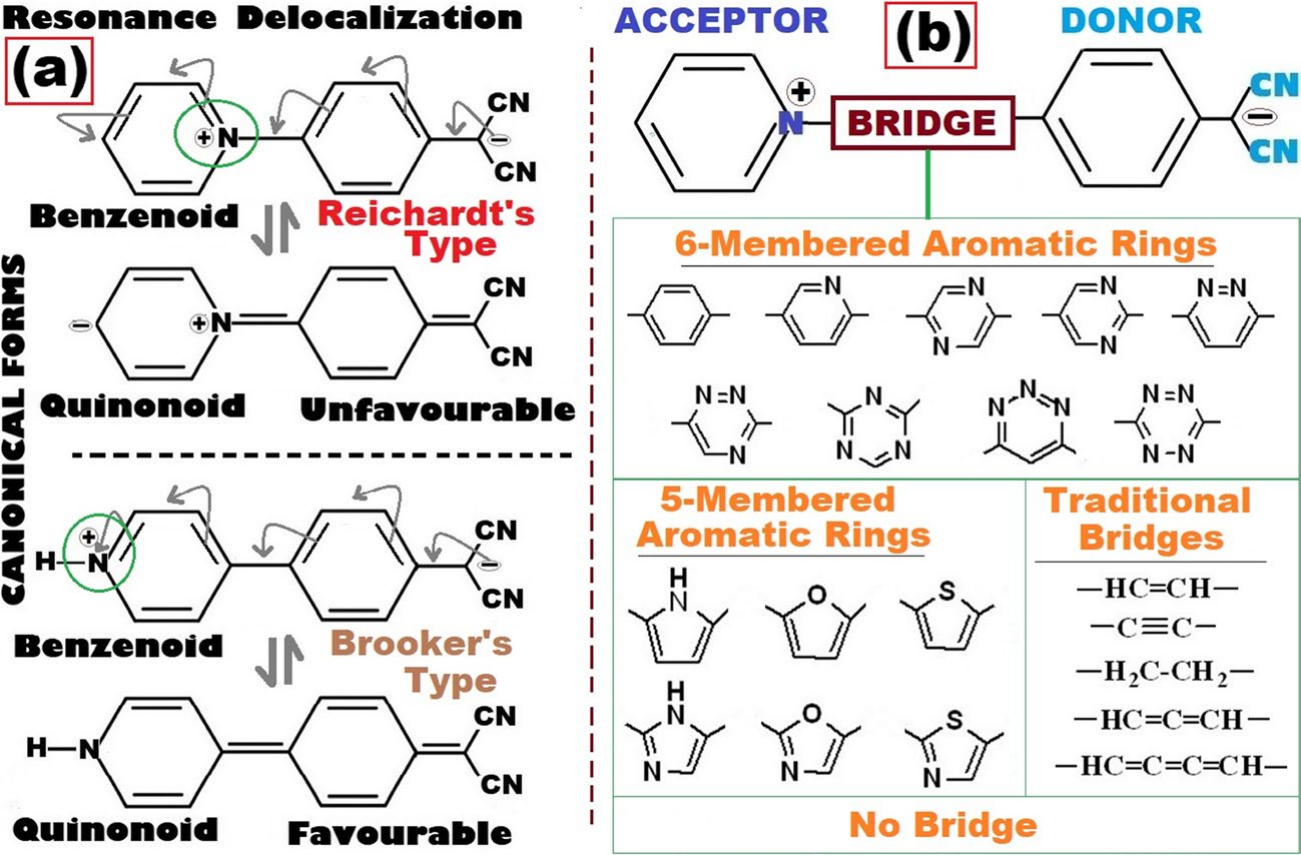
**a** Important canonical forms with the mesomeric resonance assisted delocalization of Reichardt’s and Brooker’s types of zwitterions, portraying the concept of metamerism. **b** Donor-bridge-acceptor (D-B-A) types of push–pull organic Reichardt’s types of zwitterions, with pyridinium acceptor and *p-*phenylene dicyanomethanide donor, with various bridges

The concept of metamerism belongs to the class of structural or constitutional isomerism and is well-known in organic chemistry. Like other constitutional isomerism, isomers of this class (generally referred as metamers) are also isoelectronic and have equal numbers of σ- and π-bonds (natures of bonding are conserved) but differ from other with respect to the altered connectivity at specific bonding sites. In some recent works, the concept of metamerism was investigated to account for the conformational preferences shown by zwitterions, and its impacts on various functional molecular properties [1–4]. As reported in a recent work on zwitterions [5], due to such altered connectivity, a Reichardt type [6] (inter-ring bonding mode through N-atom of the pyridinium) can adopt a twisted conformation, and a Brooker type [7] (inter-ring bonding mode through C-atom of the pyridinium) can adopt a fully planar conformation. In some of our recent works, we reported nonlinear optical (NLO) properties of series of Brooker’s types of zwitterions, where the cationic N-methyl pyridinium (to get the Brooker types) acceptors (A) and the anionic donors (D), either the para-substituted phenylene dicyanomethanides or the phenolates, were coupled through a series of monocyclic aromatic bridges [8, 9]. By comparing our reported computational results [8, 9], with some already reported experimental data [10–22], we have shown how the aromatic bridges play very significant roles on structure–property correlations in such kinds of zwitterions. In view of some of these previous reports on Brooker’s types of zwitterions [8, 9] and impacts of (non)aromatic bridges [23, 24], in this contribution, we have investigated NLO behaviors of a series of Reichardt’s types of zwitterions, instead of Brooker’s types as reported in earlier works. Molecules considered for this work are schematically presented in Scheme 1.

Scheme 1(a) shows a graphical representation of the concept of metamerism (resonance delocalization paths and relative stabilities of canonical forms obtained from the mesomeric resonance delocalization are shown). Scheme 1(a) also clearly highlights the differences in connectivity exhibited by the Reichardt and Brooker types with respect to specific bonding sites at the pyridinium acceptor side. With the same donor and bridges, in our previous work, NLO responses of only Brooker’s types of zwitterions were reported [8]. In this work, focus is completely on the Reichardt type of zwitterions (Scheme 1(b)) and compared with the previously reported Brooker types [8]. For the Reichardt types of zwitterions considered here (Scheme 1(b)), (i) p-phenylene dicyanomethanides were used as the donors [8], (ii) a series of bridges like our previous reports [8, 9, 23, 24], and (iii) pyridinium as the acceptors bonded to these bridges in Reichard’s mode (instead of the Brooker mode as reported in our earlier works [1–5]). Bridges considered here (Scheme 1(b)) can be broadly classified as aromatic bridges (six-membered and five-membered monocyclic aromatic rings) and nonaromatic bridges (including σ-, π-, allenic/cumulenic with orthogonal π-orbital types of bridges). These nonaromatic bridges were well-known and widely investigated in numerous earlier studies for various applications and are used here to make comparative assessments of the performances of the aromatic bridges. Also, a D-A directly connected molecule (without any bridge) is investigated to have a comparative assessment of the performances of the aromatic bridges.

About the significances of Reichardt’s types of zwitterions, which can be easily synthesized by simple coupling reactions [6], numerous studies for various applications can be found in the literature. For example, they are suggested as polarity indicators [25–28], as fluorescent probes for macromolecules [29], as biomarkers [30], as photosensitizers for photodynamic treatment [31], and as dyes for solar cells [32, 33]. They are also known to be useful as materials for photonic applications with their significant second order NLO responses (first hyperpolarizabilities, *β*) [1–3, 8–17, 34–49]. In most of these earlier works, focuses were either on the modulations of acceptor and/or donor groups or on the extended π-conjugations, but rarely on the systematic studies on aromatic rings as bridges [1–3, 8–17, 34–49]. In this work, with the various monocyclic aromatic bridges, 27 Reichardt’s types of zwitterions were constructed, and their detailed structure–property correlations were investigated. Dipole moment (*μ*), polarizabilities (*α*), hyperpolarizabilities (*β*), and other related properties that are useful in the domain of nonlinear optics were investigated in this work. As all these molecules were investigated for their potential applications in the field of NLO, hence a related concise discussion is provided below.

Field of NLO is vast and enriched with a massive number of research works. Materials showing NLO responses, like the molecular first hyperpolarizabilities (*β*) or bulk second-order nonlinear optical response (${\chi }^{2}$), are generally suggested to have possible applications in optical and electro-optical devices [50–59]. As research related to NLO deals with understanding of fundamental structure–property correlations of chromophores, hence such studies are often found to have applications to various other important as well as fundamental areas of chemistry and material science. With the vast literature available in the field of NLO, one can clearly see that the efficient design principles proposed by earlier researchers not only make it easy but also able to predict or design efficient new molecular NLO-phores, with remarkable accuracies [60–79]. For NLO-active molecular chromophores, the molecular first hyperpolarizability (*β*) components are usually expressed as a third-rank tensor (typically as a 3 × 3 × 3 cubic matrix) [10, 56–59]. But with the applications of restrictions due to Kleinman symmetry requirements, out of 27 usually, only 10 nonvanishing components are obtained [10, 56–59]. From here, structural or conformational manipulation techniques are only useful to reduce those 10 components further to fewer nonzero components. As the tensors have third-order characteristics, hence structural manipulation with reducing dimensionality (either making the chromophore to adopt a fully planar or pseudo-linear or fully linear configuration) is generally used to get even fewer nonvanishing components. One of the advantages associated with the above process is that it enforces the molecular chromophore to exhibit almost unidirectional charge transfer path. At the same time, intensity of charge transfer for such characteristic unidirectional charge transfer process is generally boosted with the stronger D/A combinations, like the zwitterionic types of molecular chromophores. Due to the above reasons, zwitterions were subjected to many NLO research activities in the past, and due to the same reason, this work is also focused on zwitterions. As many other fundamental aspects are also reported here, hence the current study may be useful for other areas of chemistry, physics, and material science.

## Computational methods

All the 27 molecules considered for this study (Scheme 1(b)) were fully optimized (without imposing any symmetry restrictions) using HF [80], B3LYP [81, 82], CAM-B3LYP [83], and ωB97xD [84, 85] methodologies and with 6-31G(d,p) basis set. All computations were carried out with Gaussian 09 program [86]. For few cases, aug-cc-pVDZ was used to see the impacts of basis set on structure–property correlations. For each case, vibrational frequencies were computed and analyzed to confirm these zwitterions as true minima (all the 27 molecules showed positive frequencies or no negative eigenvalues in the Hessian). In some of our recent reports, we have observed that the above-mentioned methodologies are not only suitable in accounting the charge separated characteristics of zwitterionic efficiently but also suitable in the prediction of NLO characteristics of the zwitterions [1–4, 8, 9, 87]. Moreover, in many earlier studies, some of the above-mentioned methodologies were investigated (to account for the effects of electron correlations and/or long-range corrections) to compute NLO properties, like the hyperpolarizabilities, and suggested as appropriate methodologies for such kinds of investigations [88–93]. Then, using the optimized geometry of each zwitterion, in respective methodologies, further computations were carried out. Time-dependent (TD) [94] adiabatic vertical excitation computations were performed to get various spectroscopic characteristics. Electron population computations were performed to obtain molecular frontier molecular orbitals (electron populations densities were visualized with GaussView to generate these orbitals). From the three components of the dipole moment (*μ*_*x*_, *μ*_*y*_, and *μ*_*z*_), the total resultant dipole moments were computed using the (1). Then, coupled-perturbed (CPHF) [95, 96] computations were performed to get the tensorial components of the response properties like the polarizabilities and hyperpolarizabilities (a concise description of CPHF method is provided in the supporting information). In general, six tensorial components of polarizability (*α*_*xx*_, *α*_*xy*_, *α*_*yy*_, *α*_*xz*_, *α*_*yz*_, and *α*_*zz*_) and 10 tensorial components of hyperpolarizability (*β*_*xxx*_, *β*_*xxy*_, *β*_*xyy*_, *β*_*yyy*_, *β*_*xxz*_, *β*_*xyz*_, *β*_*yyz*_, *β*_*xzz*_, *β*_*yzz*_, and *β*_*zzz*_) were obtained. Then, using these tensorial components, $\langle \alpha \rangle$(average polarizability) were computed using Eq. 2, and then, *β*_tot_ (also termed as *β*_0_: first hyperpolarizability and usually the orientationally averaged hyperpolarizabilities) was calculated using Eq. 3 [52–57]. After using the appropriate multiplication factors (*α* 1 a.u. = 0.1482 × 10^−24^ esu and *β* 1 a.u. = 8.6393 × 10^−33^ esu), obtained poalizabilities and hyperpolarizabilities were expessed in the units of esu.

1 $$\text{Total dipole moment}:{\mu }_{\text{tot}}=\sqrt{{\mu }_{x}^{2}+{\mu }_{y}^{2}+{\mu }_{z}^{2}}$$

where, *µ*_*x*_, *µ*_*y*_, and *µ*_*z*_ are the components of the dipole moments in respective directions.

2 $$\text{Average polarizability}:\langle a\rangle =\frac{1}{3}\left({a}_{xx}+{a}_{yy}+{a}_{zz}\right)$$

where the *α*_*xx*_, *α*_*yy*_, and *α*_*zz*_ are the three contributing components in orthogonal directions.

3 $$\text{First hyperpolarizability}:{\beta }_{0}=\sqrt[2]{{\beta }_{x}^{2}+{\beta }_{y}^{2}+{\beta }_{z}^{2}}$$

where *β*_*x*_, *β*_*y*_, and *β*_*z*_ components were calculated using the following equations.

4 $${\beta }_{x}= {\beta }_{xxx}+ {\beta }_{xyy}+ {\beta }_{xzz}$$

5 $${\beta }_{y}= {\beta }_{yyy}+ {\beta }_{yxx}+ {\beta }_{yzz}$$

6 $${\beta }_{z}= {\beta }_{zzz}+ {\beta }_{zxx}+ {\beta }_{zyy}$$

## Results and discussions

### No-bridge case (D-A directly connected)

For the no-bridge case as indicated in Scheme 1, the related computed data and molecular structure of the molecule (Molecule 1) are shown in Table 1. A detailed analysis for this molecule is presented here, as this molecule will be frequently used to comparatively assess the impacts of various bridges, along with comparison with some earlier literature. While only the computed ωB97xD and CAM-B3LYP data are shown in Table 1, computed data with other methodologies (HF and B3LYP) are provided as supporting information data. All the methodologies predicted twisted conformation for Molecule 1; for example, the inter-ring twisting observed at the D-A junction was found to be + 37.6° in ωB97xD methodology. Without the dispersion corrections (ωB97x/6-31G(d,p)), + 38.0° twisting was observed (a difference of 0.4°). Similarly with other basis set, not many differences in the twisting were observed in respective methodologies (example: ωB97xD/aug-cc-pVDZ twisting was + 37.9°). This clearly indicates that the basis set has no significant effects on the overall conformations of the molecules. Among the methodologies, largest and lowest values of twisting were observed with HF and B3LYP respectively, and this behavior was observed with both 6-31G(d,p) and aug-cc-pVDZ basis sets. Two recent works where the Brooker-type metamer of Molecule 1 was reported are also shown in the same table (Table 1) (Molecule 1B [3] and Molecule 1BM [8]) for comparative analysis of the structure–property correlations. To be noted is that in both the earlier reports, reported data for Molecule 1B were with 6–31 +  + G(d,p) basis set. Note: the same work reports also the data for Molecule 1 with 6–31 +  + G(d,p) basis set [3] and Molecule 1BM (reported data similar to Molecule 1, computed with 6-31G(d,p) basis set) [8] was reported to have fully planar conformations. It will be interesting to see how the planar Brooker conformation vs twisted Reichardt conformation affects the molecular properties.

**Table 1:** Optimized geometry, ground state dipole moment (*μ*_*g*_) and ground to excited state dipole difference (*Δμ*_*ge*_) in Debye, excitation energy (*ΔE*_*ge*_) in eV, absorption maximum (*λ*_*max*_) in nm, average polarizability (*<α>***)** in 10^-24^ esu**,** 1^ST^ hyperpolarizability (*β*_*0*_) in 10^-30^ esu, oscillator strength *f*_*ge*_ (unitless), coefficient of maximum contributing transition *C*_*i*_ (unitless) with frontier molecular orbitals, of Molecule 1, computed using various methodologies. Similar data for Molecule 1B [3] and Molecule 1BM [8] were also shown for comparison (ωB97xD/6-31++G(d,p) and ωB97xD/6-31G(d,p) methodologies respectively). For Molecule 1, all the data inside the () are from Reference [3] computed using ωB97xD/6-31++G(d,p)

**Molecule 1**		**Molecule 1B [3]**				**Molecule 1BM [8]**		
**Molecule 1/ Methodologies**	***μ***_***g***_	***<α>***	***β***_***0***_	***Δμ***_***ge***_	***ΔE***_***ge***_	***λ***_***max***_	***f***_***ge***_	***C***_***i***_
**CAM-B3LYP/6-31G(d,p)**	19.4	37.3	184.7	28.5	2.1	567.4	0.68	0.70
**ωB97xD/6-31G(d,p)**	20.3	36.5	215.0	11.5	2.2	563.9	0.64	0.69
**(ωB97xD/6-31++G(d,p))**	(21.2)	(40.1)	(257.2)	(--)	(--)	(583.5)	(0.60)	(0.70)
For all the methodologies transitions were always from HOMO to LUMO.	HOMO			**→**		LUMO		
**Molecule 1B [3]**	20.3	40.9	67.2	--	--	447.0	1.31	0.70
**Molecule 1BM [8]**	20.9	40.2	77.0	9.5	2.8	441.3	1.39	0.70

As shown in Table 1, for Molecule 1, the total ground state dipole moment (*µ*_*T*_ = *µ*_*g*_) was found to be 20.3 D (Debye), with *x*-component of dipole vector as the only contributor to *µ*_*g*_. Without the dispersion corrections (ωB97x/6-31G(d,p)), observed dipole moment was 0.8 D enhanced (*µ*_*T*_ = 21.1 D). Comparing Molecule 1 with Molecule 1B (metamer of Molecule 1 and its Brooker’s type zwitterion) or even with Molecule 1BM (methylated derivative of the Brooker type metamer), the dipole moments were found to be not much different from each other. Now, for Molecule 1, about the effects of basis set, like 6-31G(d,p) compared to 6–31 +  + G(d,p) values (presented inside the bracket), we were able to see slightly enhanced values of dipole moment (slightly different characteristics were also observed with all computed functional properties). Similarly with other basis set, not many differences in the dipole moments were observed in respective methodologies (examples: ωB97xD/aug-cc-pVDZ, *µ*_*T*_ = 20.9 D, and CAM-B3LYP/aug-cc-pVDZ, *µ*_*T*_ = 20.1 D). This clearly indicates that the basis set has no significant effects on the ground state dipole moments of Molecule 1. Computed hyperpolarizabilities (*β*_0_) values for Molecule 1 were found to be 215.0 × 10^−30^ esu in ωB97xD/6-31G(d,p) (enhanced value of 257.2 × 10^−30^ esu observed with 6–31 +  + G(d,p) basis set) and slightly reduced value of 184.7 × 10^−30^ esu in CAM-B3LYP/6-31G(d,p). About the effects of dispersion, when ωB97xD/6-31G(d,p) was compared with ωB97x/6-31G(d,p), for the latter cases, *β*_0_ was found to be 242.5 × 10^−30^ esu. Now, with the aug-cc-pVDZ basis set, enhanced values of *β*_0_ were observed with respect to each methodology (examples: ωB97xD, *β*_0_ = 253.0 × 10^−30^ esu, and CAM-B3LYP, *β*_0_ = 228.4 × 10^−30^ esu). In all the methodologies, Molecule 1 was found to be showing *β*_*x*_ as the only contributing component (with *β*_*xxx*_ as the main contributing tensor). Now, comparing ωB97xD/6-31G(d,p) results of Molecule 1, with Molecule 1B or Molecule 1BM, highly enhanced values of *β* were observed for Molecule 1 (3.8 times enhanced compared to 1B and 2.8 times enhanced compared to 1BM). Enhancement in hyperpolarizability observed for Molecule 1 (Reichardt’s type) compared to the other two metamers (Brooker’s type) is quite interesting. Thus, it can be used as manipulation technique to obtain efficient NLO-phores, through the induction of metameric effects [1–4]. Average polarizability of Molecule 1 (ωB97xD/6-31G(d,p): < *α* >  = 36.5 × 10^−24^ esu) was found to be like its metameric Brooker type (Molecule 1B). Like the dipole moment, polarizability was also found to be not getting much affected by the metameric manipulation. Vertical excitation study of Molecule 1 (ωB97xD/6-31G(d,p) methodology) showed Δ*µ*_*ge*_ as 11.5 D (lower value of ground to excited state adiabatic dipole difference indicates a nonreversal of charge localized state during excitation) and *λ*_max_ as 563.9 nm (with oscillator strength, *f*_ge_ = 0.64). Now, comparing with its metamer (Molecule 1B or even Molecule 1BM), Molecule 1 was found to be showing a highly red-shifted absorption with relatively lower oscillator strength. Not only this is interesting, but also it is important as it clearly highlights the important role played by metamerism (Brooker’s vs Reichardt’s types of zwitterions). For Molecule 1, the excitation process was found to be associated with HOMO to LUMO (highest occupied and lowest unoccupied molecular orbitals respectively) transition, and both the orbitals were found to be delocalized in natures (with relatively more localized electron densities at the D-side in HOMO and at the same time at the A-side in LUMO).

Now, comparing ωB97xD/6-31G(d,p) results of Molecule 1 (Table 1) with those of the other methodologies with the same basis set (Table S1: SI Text), major differences in hyperpolarizabilities were observed. ωB97xD predicted the highest value and B3LYP predicted the lowest value of *β* (88.9 × 10^−30^ esu) among all the methodologies. Enhanced value of *β* was observed for HF (120.5 × 10^−30^ esu) and CAM-B3LYP (184.7 × 10^−30^ esu) compared to B3LYP methodologies. Trends in excitation behaviors showed that the HF predicted exceptionally lower absorption maximum compared to all the DFT methodologies (all DFT methodologies show nearly equal absorption wavelengths). Now, in the next sections, the molecules considered are similar zwitterionic molecules, where either aromatic or nonaromatic bridges (mostly the well-known traditional bridges) are introduced in between the same D and A of Molecule 1. Based on the observations for Molecule 1, for the rest of the systems, computations were carried out mainly with 6-31G(d,p) basis set combined with all the four methodologies. The comprehensive analysis presented in this section for Molecule 1 is often used to compare with other molecules to qualitatively as well as quantitatively assess the impacts of aromatic bridges on the structure–property correlations.

### Impacts of aromatic bridges

Like our previous studies on Brooker’s types of zwitterions [8, 9], here, also roles of aromatic rings as bridges were investigated with a series of monoaromatic rings (many six-membered and five membered rings). In general, for six-membered aromatic rings as bridges, D and A were connected to the bridges, in the para-positions with respect to each other. On the other hand, for the cases with five-membered aromatic rings as the bridges, D and A were connected to the bridges in such ways that the distances between D and A were always maximized. Many times, results from this study are compared with those earlier works on Brooker’s types [8, 9] to assess the impacts of aromatic bridges with the Reichardt type of zwitterions.

#### Benzene ring as the bridge

The Reichardt type zwitterion with benzene ring as the bridge, i.e., Molecule 2, and its optimized geometry along with computed properties are shown in Table 2 and Table S2 (ωB97xD and CAM-B3LYP data in Table 2, and HF and B3LYP data in Table S2 – SI Text). Unlike Molecule 1 (only one inter-ring junction), here in Molecule 2, due to the presence of the bridge between the D and A, there were two inter-ring junctions (one at the D-side and one at the A-side). In the twisted conformation of Molecule 2, twisting of + 39.9° and − 19.6° was observed at the A-side and D-side junctions respectively (positive and negative values indicate that the relative twisting was in the opposite direction). Without the dispersion corrections (ωB97x/6-31G(d,p)), + 42.0.0° (A-side) and − 18.6 (D-side), twist angles were observed (slightly different when dispersion effects were considered). Similarly with other basis set, not many differences in the twisting were observed in respective methodologies (example: ωB97xD/aug-cc-pVDZ twisting angles were found to be + 37.0° in the A-side and + 10.1° in the D-side). This clearly indicates that the basis set has minimal effects on the overall conformations of the molecules. Among the methodologies, the largest and lowest values of twisting were observed with HF and B3LYP respectively, showing similar trends of performance of methodologies like that of Molecule 1 (same trends were observed with both 6-31G(d,p) or aug-cc-pVDZ basis set considerations). Now, one can see that the relative twisting between the A-side and D-side, the former showed a larger twisting compared to the latter. This may be due to possible partially stabilizing resonance delocalization happening at the D-side of the zwitterion.

**Table 2:** Optimized geometry, ground state dipole moment (*μ*_*g*_) and ground to excited state dipole difference (*Δμ*_*ge*_) in Debye, excitation energy (*ΔE*_*ge*_) in eV, absorption maximum (*λ*_*max*_) in nm, average polarizability (*<α>***)** in 10^-24^ esu**,** first hyperpolarizability (*β*_*0*_) in 10^-30^ esu, oscillator strength *f*_*ge*_ (unitless), coefficient of maximum contributing transition *C*_*i*_ (unitless) with frontier molecular orbitals, of Molecule 2, computed various methodologies

**Molecule 2 / Methodologies**	***μ***_***g***_	***<α>***	***β***_***0***_	***Δμ***_***ge***_	***ΔE***_***ge***_	***λ***_***max***_	***f***_***ge***_	***C***_***i***_
**CAM-B3LYP/6-31G(d,p)**	30.8	78.8	869.7	18.0	1.5	805.9	0.95	0.71
**ωB97xD/6-31G(d,p)**	33.5	74.3	1291.2	45.6	1.6	799.6	0.85	0.70
	HOMO			**→**	LUMO			

Results of Molecule 2 were then compared with Molecule 1, to assess the impact of phenylene bridge and, with the literature reported Brooker’s metamer also, to assess the impact of metamerism on the functional molecular properties [8]. Now, compared to Molecule 1, significant enhancements in various molecular properties were observed for Molecule 2, for example, dipole moment of 33.5 D (Molecule 2) compared to 20.3 D (Molecule 1), and like Molecule 1, for Molecule 2, also the dipole moment was found to be with only one contributing tensor (*x*-direction). Like the dipole, polarizability of Molecule 2 (< *α* >  = 74.3 × 10^−24^ esu) was found to around double that of Molecule 1 (< *α* >  = 36.5 × 10^−24^ esu). Now, about first hyperpolarizability (*β*_0_) for Molecule 2 (*β*_0_ = 1291.2 × 10^−30^ esu), it was found to be approximately 6 times enhanced compared to Molecule 1 (*β*_0_ = 215.0 × 10^−30^ esu). With aug-cc-pVDZ basis set, as expected enhanced values of hyperpolarizabilities were observed (for example, CAM-B3LYP: 1110.8 × 10^−30^ esu in aug-cc-pVDZ, compared to 869.7 × 10^−30^ esu in 6-31G(d,p) basis set), and exactly similar trends were observed with Molecule 1. Not only that the above functional properties of Molecule 2 were found to be unidirectional (*x*-direction), like that of Molecule 1, but also, one can clearly see the impact of the *p*-phenylene bridge on various response properties, when compared with Molecule 1. Now, when these properties of Molecule 2 were compared with its Brooker metamer (nearly planar conformation and *μ*_*g*_ = 32.2 D, < *α* >  = 81.5 × 10^−24^ esu, and *β*_*0*_ = 572.2 × 10^−30^ esu) [8], one can see that although nearly similar values of dipole moment as well as polarizabilities were observed, but hyperpolarizability for Molecule 2 was found to be 2.3 times enhanced than its metamer. This comparative analysis clearly highlights the impact of metamerism on structure–property correlations of such kinds of zwitterions. This also shows that the Reichardt type is a better chromophore (NLO-phore) than the Brooker type of zwitterion (the same trend was observed with Molecule 1). To have an idea about the reason for better performance of Molecule 2 as NLO-phore compared to Molecule 1, we took the help of the well-known qualitative two-state model (which accounts for only the ground and first excited states) as shown in Eq. 7 [10, 56].

7 $$\beta \approx \frac{{f}_{\text{ge}}{\Delta \mu }_{\text{ge}}}{{\Delta E}_{\text{ge}}^{3}}$$

With the help of the two-state model, one can see that compared to Molecule 1, Molecule 2 showed enhanced values of *f*_ge_, Δ*µ*_ge_ and a large, red-shifted absorption (lower value of Δ*E*_ge_). The combined effects of all these three can significantly affect the enhanced hyperpolarizability of Molecule 2 than Molecule 1. One can also see that Δ*E*_ge_, which is in the denominator (as a cubic term: Eq. 7), the decrease in the value for Molecule 2 compared to Molecule 1, might be the leading factor in significantly enhancing the *β* value. Now, analysis of the vertical excitation for Molecule 2 showed the associated transition dipole moment (Δ*µ*_ge_ = 45.6 D, which is adiabatic dipole difference between the ground and excited states) to be quite large, even compared to the ground state dipole moment (*µ*_*g*_ = 33.5 D). This clearly indicates the reversal of dipole direction upon excitation, thus indicating a stronger charge transfer during excitation process. When compared with Molecule 1, stronger charge transfer characteristics of Molecule 2 were observed. As a consequence of such charge transfer characteristics, the oscillator strength of Molecule 2 (*f*_ge_ = 0.85) was found to be more prominent compared to Molecule 1 (*f*_ge_ = 0.64). Now, compared to Molecule 1 (*λ*_max_ = 563.9 nm), Molecule 2 showed largely red-shifted absorption maximum (*λ*_max_ = 799.6 nm). All these observations again clearly highlight the importance of aromatic (benzene ring) bridge in structure–property correlations. Now, comparing Molecule 2 with its metamer reported in literature [8], one can see that the absorption is red shifted (compared to *λ*_max_ = 614.2 nm), oscillator strength was found to be weaker (compared to *f*_ge_ = 1.92), but has largely enhanced hyperpolarizability. This is quite interesting, as it clearly highlights the important role played by the metamerism (Brooker’s vs Reichardt’s types of zwitterions) and outlines the importance of NLO-phore or in general chromophore design technique with extremely minimal structural manipulations with metamerism. Now, for Molecule 2, the excitation was found to be associated with HOMO to LUMO transition, and both the orbitals were found to be delocalized in nature. Also compared to Molecule 1, relatively more localized electron densities at the D-side in HOMO and at the A-side in LUMO were observed for Molecule 2, again clearly indicating the role of aromatic benzene ring as the bridge in the charge transfer path between the D and A.

When the performances of methodologies were analyzed (Table 2 and Table S2—SI Text), we observed some differences in the quantitative values of the molecular properties discussed previously (but the observed trend was very much like Molecule 1). While HF predicted the largest value of dipole moment (41.3 D), at the same time, B3LYP predicted the lowest value (25.0 D). Now, about the polarizability, while HF showed a very low value (43.1 × 10^−24^ esu), at the same time, relatively larger values of polarizabilities (like those of ωB97xD) were observed for other DFT methodologies (for example, B3LYP: 79.4 × 10^−24^ esu). For Molecule 2, ωB97xD predicted a larger value of hyperpolarizability than all other methodologies (HF: 377.2 × 10^−30^ esu, B3LYP: 208.4 × 10^−30^ esu, and CAM-B3LYP: 869.7 × 10^−30^ esu. Analysis of excitation studies indicates that HF showed exceptionally lower absorption maximum (485.0 nm compared to ωB97xD, 799.6 nm) than all the DFT methodologies (they all show nearly equal absorption wavelengths).

#### Pyridine as the bridge

With pyridine ring as the bridge, two molecules, Molecule 3a (pyridine N-atom near A-side) and Molecule 3b (pyridine N-atom near D-side), were investigated. Along with their optimized geometries, computed properties are shown in Table 3 (results from HF and B3LYP are provided in Table S3: supporting information). Now, due to N-atoms in the pyridine bridges, CH…N type of hydrogen bonding interactions, either in the D-side or the A-side rings, can be expected. As a result, Molecule 3a showed a lesser twisted conformation (twisting angles, − 17.0° in the D-side and + 22.70° in the A-side). At the same time, Molecule 3b planar (− 0.3° twisting) at the D-side and a twisted conformation (+ 38.9° twisting) at the A-side. Thus, it was observed that for CH…N hydrogen bonding when closer to the D-site, it was found to be more influential in exerting planarity than when it is close to the A-site. This clearly signifies the importance of the heteroatom positions in the bridges on conformational preferences shown by such types of zwitterions.

**Table 3:** Optimized geometries, ground state dipole moments (*μ*_*g*_) and ground to excited state dipole differences (*Δμ*_*ge*_) in Debye, excitation energies (*ΔE*_*ge*_) in eV, absorption maxima (*λ*_*max*_) in nm, average polarizabilities (*<α>***)** in 10^-24^ esu**,** first hyperpolarizabilities (*β*_*0*_) in 10^-30^ esu, oscillator strengths *f*_*ge*_ (unitless), coefficients of maximum contributing transition *C*_*i*_ (unitless) with frontier molecular orbitals, of Molecules 3a & 3b, computed using CAM-B3LYP and ωB97xD methodologies

**Molecule #**	**Methodologies**	***μ***_***g***_	***<α>***	***β***_***0***_	***Δμ***_***ge***_	***ΔE***_***ge***_	***λ***_***max***_	***f***_***ge***_	***C***_***i***_
**Molecule 3a**	**CAM-B3LYP**	29.5	76.1	801.7	42.3	1.6	779.3	0.98	0.71
	**ωB97xD**	31.4	72.3	1128.6	18.8	1.7	737.5	1.01	0.70
	HOMO				**→**	LUMO			
**Molecule 3b**	**CAM-B3LYP**	29.8	75.7	723.7	17.2	1.6	754.6	1.00	0.71
	**ωB97xD**	32.0	72.3	977.0	44.2	1.7	737.4	0.95	0.69
	HOMO				**→**	LUMO			

To account for the impacts of the heteroatoms in the bridge, results of Molecules 3a and 3b were compared with Molecule 2. To account for the impacts of metamerism, Molecules 3a and 3b were compared with literature reported their corresponding Brooker metamers [8]. Compared to the dipole moment of Molecule 2 (33.5 D), no significant changes were observed (Molecule 3a: 31.4 D and Molecule 3b: 32.0 D). Here also, like the previous cases, dipole moments were found to have one contributing tensor in the *x*-direction. Both the molecules showed equal polarizabilities (< *α* >  = 72.3 × 10^−24^ esu) and closer to Molecule 2 (< *α* >  = 74.3 × 10^−24^ esu). About the first hyperpolarizabilities (*β*_0_), both showed marginally lower values (Molecule 3a: 1128.6 × 10^−30^ esu and Molecule 3b: 977.0 × 10^−30^ esu) compared to Molecule 2 (*β*_0_ = 1291.2 × 10^−30^ esu), but at the same time, around 4.5–5.3 times enhanced compared to Molecule 1 (*β*_0_ = 215.0 × 10^−30^ esu). Now, between Molecules 3a and 3b, it is interesting to observe that Molecule 3a (pyridine N-atom: A-side) showed relatively larger hyperpolarizability than Molecule 3b (pyridine N-atom: D-side). This is definitely useful in the fine-tuning of optimal chromophore design (recently corresponding Brooker’s type of zwitterions was also reported to be exhibiting the same kind of behavior [8, 9]). When Molecules 3a and 3b were compared with their respective Brooker metamers [8], while similar behaviors were observed for the dipole moments and polarizabilities, at the same time, reasonable differences were observed with the hyperpolarizabilities (*β*_0_ values 611.2 × 10^−30^ esu and 426.2 × 10^−30^ esu for the corresponding Brooker metamers of Molecules 3a and 3b respectively). The observed enhanced hyperpolarizabilities for Reichardt’s over Brooker’s types again clearly highlights the impact of metamerism on structure–property correlations.

Analyses of the vertical excitations, Molecules 3a and 3b were found to be showing very similar characteristics. The major differences observed were for Δ*µ*_ge_ (18.2 D and 44.2 D respectively for Molecules 3a and 3b). Thus, the charge transfer characteristics of Molecule 3a were found to be very similar to Molecule 2, but for Molecule 3a, it was found to be quite different. Red-shifted absorption maxima were observed for Molecules 3a and 3b, compared to their respective Brooker types of metamers, already reported in literature (*λ*_max_ = 627.8 nm and *λ*_max_ = 586.4 nm for the respective Brooker metamers [8]). This observation clearly shows the impacts of metamerism (Brooker’s vs Reichardt’s types) and may be useful for suitable chromophore design. Frontier molecular orbitals participating in the excitation processes were found to be from HOMO to LUMO, for Molecules 3a and 3b. Both HOMO and LUMO were found to be delocalized in nature, with relatively more localized electron densities at the D-side in HOMO and at the A-side in LUMO.

Now, comparing various methodologies for Molecules 3a and 3b (ωB97xD and CAM-B3LYP: Table 3, and HF and B3LYP methodologies: Table S3 – SI Text), although very similar trends were observed like as shown by Molecule 1 and 2, but some noticeable differences in the quantitative values were observed. HF predicted the largest values and B3LYP predicted the lowest values of dipole moments. Related to polarizability, while HF showed very low values (45.7 and 46.7 × 10^−24^ esu respectively, for Molecules 3a and 3b), at the same time, relatively larger values of polarizabilities (like those of ωB97xD) were observed for other DFT methodologies. For both the molecules, ωB97xD predicted larger values of hyperpolarizabilities compared to other methodologies, and at the same time, HF predicted the very low values of absorption maxima, compared to all the methodologies.

#### Diazines as the bridges

With diazine bridge cases (with two N-atoms in the aromatic rings as the bridges), four molecules were investigated, where Molecule 4 has 1,4-diazine or pyrazine as the bridge; Molecule 5 has 1,2 diazine or pyridazine as the bridge; and Molecules 6a and 6b have 1,3-diazine or pyrimidine as the bridges (6a: two N-atoms closer to A-side, and 6b: two N-atoms closer to D-side). Along with their optimized geometries, computed properties of all four molecules are shown in Table 4 (results from HF and B3LYP are provided in supporting information: Table S4).

**Table 4:** Optimized geometries, ground state dipole moments (*μ*_*g*_) and ground to excited state dipole differences (*Δμ*_*ge*_) in Debye, excitation energies (*ΔE*_*ge*_) in eV, absorption maxima (*λ*_*max*_) in nm, average polarizabilities (*<α>***)** in 10^-24^ esu**,** first hyperpolarizabilities (*β*_*0*_) in 10^-30^ esu, oscillator strengths *f*_*ge*_ (unitless), coefficients of maximum contributing transition *C*_*i*_ (unitless) with frontier molecular orbitals, of Molecules 4, 5, 6a & 6b, computed using CAM-B3LYP and ωB97xD methodologies

**Molecule #**	**Methodologies**	***μ***_***g***_	***<α>***	***β***_***0***_	***Δμ***_***ge***_	***ΔE***_***ge***_	***λ***_***max***_	***f***_***ge***_	***C***_***i***_
**Molecule 4**	**CAM-B3LYP**	27.5	72.7	625.8	40.9	1.8	683.7	1.23	0.71
	**ωB97xD**	29.2	70.4	799.0	42.4	1.9	667.1	1.22	0.69
	HOMO				**→**	LUMO			
**Molecule 5**	**CAM-B3LYP**	28.6	73.4	714.7	41.2	1.7	732.5	1.02	0.71
	**ωB97xD**	30.5	70.0	924.8	42.7	1.7	708.9	1.00	0.69
	HOMO				**→**	LUMO			
**Molecule 6a**	**CAM-B3LYP**	27.7	73.0	802.0	40.8	1.7	725.4	1.11	0.71
	**ωB97xD**	29.7	69.7	1053.9	42.5	1.8	707.0	1.08	0.70
	HOMO				**→**	LUMO			
**Molecule 6b**	**CAM-B3LYP**	29.5	72.9	672.9	41.9	1.6	730.8	0.99	0.71
	**ωB97xD**	31.4	69.7	851.4	43.4	1.8	708.0	0.96	0.69
	HOMO				**→**	LUMO			

Due to the two N-atoms of the bridges, resulting in multiple CH…N types of hydrogen bonding interactions, although twisted conformations (most often at A-side junctions) were observed, but the extents of twisting were found to lower than the pyridine bridge cases. Earlier work related to Brooker’s types of zwitterions with the same kinds of bridges were reported to be in planar conformations [8, 9]. This clearly highlights the importance of the multiple heteroatoms in Reichardt’s types of zwitterions. About the dipole moments, all the four molecules showed reasonably good values and are comparable to Molecule 2 (or even compared to Molecules 3a and 3b). Like the previous discussed molecules, all four showed only one contributing tensor (*x*-component) to the total ground state dipole moment (Molecule 5 was found to be showing some contribution from the *µ*_*y*_ tensor, example: CAM-B3LYP: *µ*_***y***_ = 3.27 D). Compared to Molecule 1, while all the four showed enhanced polarizabilities (*α*), thus indicating the impacts of diazine bridges on polarizabilities, at the same time, compared to Molecule 2 (or even compared to Molecules 3a and 3b), no significant differences were observed. Similar behaviors were also observed with hyperpolarizabilities. Enhanced values of *β* were observed compared to Molecule 1, but comparable to benzene (slightly lower value) and pyridine bridge cases. For Molecules 6a and 6b, similar behaviors were observed like those of Molecules 3a and 3b (larger *β* with N-atoms of the pyrimidine bridge closer to A-side, compared to when they were closer to D-side). When these four molecules were compared with their respective Brooker types [8], while no significant differences were observed in dipole moments and polarizabilities, but for hyperpolarizabilities, enhanced values were observed (Molecule 4: 799.0 × 10^−30^ esu compared to 415.8 × 10^−30^, Molecule 5: 924.8 × 10^−30^ esu compared to 527.9 × 10^−30^, Molecule 6a: 1053.9 × 10^−30^ esu compared to 665.1 × 10^−30^, and Molecule 6b: 851.4 × 10^−30^ esu compared to 388.3 × 10^−30^). The observed enhanced hyperpolarizabilities for Reichardt’s over Brooker’s types of zwitterions clearly highlight the impacts of metamerism on structure–property correlations and roles of various diazene bridges.

Electronic absorptions indicate very similar behaviors by all the four molecules (slight blue shift, with *λ*_max_ = 667.1 nm for Molecule 4, was observed compared to others). Like the previous cases, here, also excitations were found to be of HOMO to LUMO electronic transitions, and the natures of these orbitals were found to be delocalized (with partially localized electron densities at the D-sides in HOMOs and at the same time, at the A-sides in LUMOs). Analysis of the *λ*_max_ values showed red-shifted absorptions for all the four molecules compared to Molecule 1 and slightly blue-shifted absorptions compared to Molecule 2, thus, clearly highlighting the roles played by these aromatic bridges. Now, comparing with their corresponding Brooker type metamers [8], all of them showed red-shifted absorption maxima (*λ*_max_ values, for Molecule 4, 667.1 nm compared to 584.6 nm; Molecule 5, 708.9 nm compared to 614.6 nm; Molecule 6a, 707.0 nm compared to 637.9 nm; and Molecule 6b, 708.0 nm compared to 568.2 nm). This observation is also important as it shows the impacts of metamerism (Brooker’s vs Reichardt’s types) and may be useful for suitable zwitterionic chromophore design.

Comparison of the results from various methodologies for all the four molecules (Table 4 and Table S4 – SI Text), showed very similar trends like that of the previously discussed molecules, but some noticeable differences in quantitative values of molecular properties were observed. With respect to dipole moments, HF predicted the largest values and B3LYP predicted the lowest values; but when polarizabilities were compared, HF predicted low values and all DFT methodologies predicted relatively larger values (DFT values were comparable to each other). Now, about the hyperpolarizabilities, while ωB97xD predicted larger values, at the same time, B3LYP showed the lowest values of hyperpolarizabilities. Now, about absorption, while HF predicted the very low values of absorption maxima for all the four molecules, at the same time, all the DFT methodologies predicted very similar absorption maxima and larger values than HF.

#### Triazines as the bridges

For the triazine bridges (with three N-atoms in the ring), we were able to get four different molecules. Where Molecules 7a and 7b have 1,2,4-triazines as bridges (they differ in the relative positions of the N-atoms with respect D and A), Molecule 8 has 1,3,5-triazine as the bridge, and Molecule 9 has 1,2,3-triazine as the bridge. All these four zwitterions were fully optimized and their respective optimized geometries, along with their various computed molecular properties are shown in Table 5 (results from HF and B3LYP are provided in supporting information: Table S5).

**Table 5:** Optimized geometries, ground state dipole moments (*μ*_*g*_) and ground to excited state dipole differences (*Δμ*_*ge*_) in Debye, excitation energies (*ΔE*_*ge*_) in eV, absorption maxima (*λ*_*max*_) in nm, average polarizabilities (*<α>***)** in 10^-24^ esu**,** first hyperpolarizabilities (*β*_*0*_) in 10^-30^ esu, oscillator strengths *f*_*ge*_ (unitless), coefficients of maximum contributing transition *C*_*i*_ (unitless) with frontier molecular orbitals, of Molecules 7a, 7b, 8 & 9, computed using CAM-B3LYP and ωB97xD methodologies

**Molecule #**	**Methodologies**	***μ***_***g***_	***<α>***	***β***_***0***_	***Δμ***_***ge***_	***ΔE***_***ge***_	***λ***_***max***_	***f***_***ge***_	***C***_***i***_
**Molecule 7a**	**CAM-B3LYP**	26.7	69.8	673.6	39.2	1.8	688.2	1.07	0.70
	**ωB97xD**	28.2	67.4	806.6	40.8	1.9	508.6	1.13	0.69
	HOMO				**→**	LUMO			
**Molecule 7b**	**CAM-B3LYP**	27.5	70.6	602.0	40.3	1.8	683.0	1.14	0.70
	**ωB97xD**	29.0	67.9	730.4	41.6	1.9	660.2	1.13	0.69
	HOMO				**→**	LUMO			
**Molecule 8**	**CAM-B3LYP**	27.0	45.7	298.6	26.8	1.2	1015.9	0.03	0.69
	**ωB97xD**	27.4	44.8	191.6	24.9	1.5	812.0	0.03	0.68
	HOMO				**→**	LUMO			
**Molecule 9**	**CAM-B3LYP**	28.3	52.4	731.4	23.8	1.0	1170.9	0.08	0.69
	**ωB97xD**	29.0	48.8	403.2	25.3	1.4	910.7	0.07	0.68
	HOMO				**→**	LUMO			

While Molecules 7a and 7b showed linear (or pseudo-linear) arrangements of the three aryl units, at the same time, Molecules 8 and 9 showed bent or V-shaped arrangements. Molecules 7a and 8 were found to have fully planar conformations, and Molecules 7b and 9 were found to have partially twisted conformations (planar in D-sides but twisted in the A-sides. Inter-ring twisting, Molecule 7b: 20.0° and Molecule 9: 30.0°). Compared to pyridine (one N-atom) or diazine (two N-atoms) cases, here, for the triazines (three N-atoms), now, the multiple CH…N types of hydrogen bonding interactions were found to have some impacts on the inter-ring junctions and thus in their observed conformations. This is interesting as the Brooker type metamers of these zwitterions were reported to have planar conformations [8]. This clearly highlights the importance of multiple heteroatoms on conformational preferences, and their impacts even on the Reichardt types of zwitterions. Reasonably good dipole moments were observed for all the four molecules and were comparable to Molecule 2. While Molecules 7a and 7b (linear arrangements) showed only one contributing tensor (*x*-component) to their total ground state dipole moments, at the same time, Molecules 8 and 9, owing to their bent conformations, showed considerable contributions from other components (example: CAM-B3LYP: *µ*_*y*_ = 5.56 D). While polarizabilities (*α*) for Molecules 7a and 7b were comparable to those of Molecule 2, at the same time, for Molecules 8 and 9, *α* values were found to be much lower (comparable to Molecule 1). This shows the impact of triazine bridges not only on the molecular conformations but also on their dipole moments and polarizabilities.

About the hyperpolarizabilities, enhanced values of *β* were observed for Molecules 7a, 7b, and 9 (relatively lower value of *β* for Molecule 9, compared to Molecules 7a and 7b) compared to Molecule 1. Surprisingly, extremely low value of *β* was observed for Molecule 8. Regardless of the bent conformations shown by Molecules 8 and 9, all the four molecules showed *β*_*x*_ as the major contributing component to the *β*_0_. We then compared these molecules with their respective Brooker type metamers [8]. Compared to their respective Brooker types, some minor differences (usually slightly enhanced) were observed with the dipole moments and polarizabilities, but very significant differences in hyperpolarizabilities were observed (Molecule 7a: 806.6 × 10^−30^ esu compared to 532.9 × 10^−30^ esu, Molecule 7b: 730.4 × 10^−30^ esu compared to 414.8 × 10^−30^ esu, Molecule 8: 191.6 × 10^−30^ esu compared to 722.9 × 10^−30^ esu, and Molecule 9: 403.2 × 10^−30^ esu compared to 1224.6 × 10^−30^ esu). One can see that like the other molecules, here, also enhanced values of *β* were observed for Molecules 7a and 7b with the Reichardt metamers, but at the same time, highly enhanced values of *β* were observed for the Brooker metamers in the cases of Molecules 8 and 9. This definitely shows that for triazine bridges, when the conformations switched over from the regular linear types to 2D arrangements, Brooker’s types are more preferable over the Reichardt types. This again highlights the impacts of metamerism and natures of the bridges, on structure–property correlations in suitable chromophore design.

Vertical excitation computations showed different behaviors for Molecules 7a and 7b, than Molecules 8 and 9. Like all the previous cases, here, also the excitations of all the four molecules were found to be associated with HOMO to LUMO electronic transitions. While for Molecules 7a and 7b, these two orbitals were found to be delocalized, at the same time, localized molecular orbitals were observed for Molecules 8 and 9 (localized electron densities at the D-sides in HOMOs and at the A-sides in LUMOs). Comparably weaker absorption intensities (lower values of *f*_ge_) and lower values of Δ*µ*_ge_ were observed for Molecules 8 and 9, compared to Molecules 7a and 7b. These observations clearly show the impacts of conformational changes on molecular properties. Analysis of the *λ*_max_ values showed red-shifted absorptions for three molecules (except Molecule 7a, which showed blue shift) compared to Molecule 1. Now, at the same time, compared to Molecule 2, Molecule 7a and 7b showed blue-shifted absorptions, and Molecules 8 and 9 showed red-shifted absorption maxima. Then, all four molecules were compared with their already reported respective Brooker type metamers [8] (*λ*_max_ values, for Molecule 7a, 508.6 nm compared to 609.3 nm; Molecule 7b, 660.2 nm compared to 581.4 nm; Molecule 8, 812.0 nm compared to 1252.1 nm; and Molecule 9, 910.7 nm compared to 1225.7 nm). As observed, except Molecule 7b, in all other cases, Brooker’s type showed red-shifted absorption maxima. Especially for Molecules 8 and 9, the absorptions were in near-IR or IR regions of electromagnetic spectrum. These observed differences are important with respect to metamerism (Brooker’s vs Reichardt’s types) as well as the roles of triazine bridges on various functional molecular properties.

Then, for these four molecules, results from various methodologies (Table 5 and Table S5 – SI Text) were compared. Similar trends like those of the previously discussed molecules were observed, with some noticeable differences in the quantitative values of molecular properties (especially for the hyperpolarizability values). With respect to dipole moments, HF predicted the largest values and B3LYP predicted the lowest values. Now, when polarizabilities were compared, HF predicted low values and all the three DFT methodologies predicted relatively larger values of polarizabilities (also values were found to be comparable to each other) for Molecules 7a and 7b. But, for Molecules 8 and 9, though the same trend was found to be maintained, relatively lower values of *α* were observed with CAM-B3LYP methodology. In the case of hyperpolarizabilities, for Molecules 7a and 7b, while ωB97xD predicted larger values, at the same time, B3LYP showed the lowest values. But, for Molecules 8, the largest value of *β* was observed for B3LYP and the lowest value for HF (comparable to ωB97xD), and at the same time, for Molecule 9, again, the lowest value was shown by HF, and the largest value was observed for the CAM-B3LYP methodology. Now, about absorption characteristics, while HF predicted the very low values of absorption maxima for all the four molecules, at the same time, all the DFT methodologies predicted very different absorption maxima, especially for Molecules 8 and 9.

#### Tetrazine as the bridge

Molecule 10 (tetrazene as the bridge), with its optimized geometry and computed molecular properties, is shown in Table 6 and Table S6 (ωB97xD and CAM-B3LYP data in Table 6, and HF and B3LYP data in Table S6 – SI Text). As expected, due to four N-atoms, a fully planar conformation was observed. Ground state dipole moment (*µ*_*g*_) was found to be 27.1 D (larger than Molecule 1 and slightly lower than Molecule 2). Average polarizability (< *α* >) value was found to be 65.9 × 10^−24^ esu. Now, about the hyperpolarizability of Molecule 10 (*β*_0_ = 678.5 × 10^−30^ esu), it was found to be approximately 3 times enhanced compared Molecule 1, but almost half of the value shown by Molecule 2. About the absorption maximum of Molecule 10 (*λ*_max_ = 627.5 nm with *f*_ge_ = 1.30 and associated with HOMO to LUMO transition with fully delocalized orbitals), it was found to be blue shifted compared to Molecule 2.

**Table 6:** Optimized geometry, ground state dipole moment (*μ*_*g*_) and ground to excited state dipole difference (*Δμ*_*ge*_) in Debye, excitation energy (*ΔE*_*ge*_) in eV, absorption maximum (*λ*_*max*_) in nm, average polarizability (*<α>***)** in 10^-24^ esu**,** first hyperpolarizability (*β*_*0*_) in 10^-30^ esu, oscillator strength *f*_*ge*_ (unitless), coefficient of maximum contributing transition *C*_*i*_ (unitless) with frontier molecular orbitals, of Molecule 10, computed using CAM-B3LYP and ωB97xD methodology

**Molecule 10**	**Methodologies**	***μ***_***g***_	***<α>***	***β***_***0***_	***Δμ***_***ge***_	***ΔE***_***ge***_	***λ***_***max***_	***f***_***ge***_	***C***_***i***_
	**CAM-B3LYP**	25.9	67.9	582.9	25.9	1.9	651.6	1.24	0.71
	**ωB97xD**	27.1	65.9	678.5	27.1	2.0	627.5	1.30	0.69
	HOMO				**→**	LUMO			

Then, results of this Reichardt metamer were compared with its corresponding Brooker metamer [8]. Reported data for the Brooker metamer of Molecule 10 (planar conformation, with *μ*_*g*_ = 29.6 D, < *α* >  = 74.6 × 10^−24^ esu, *β*_0_ = 456.6 × 10^−30^ esu, and *λ*_max_ = 586.0 nm [8]), showed comparable values of dipole moment as well as polarizabilities like those of Reichardt’s metamer. But the hyperpolarizability of Molecule 10 was found to be enhanced compared to its Brooker counterpart. At the same time, absorption maximum for Molecule 10 was found to be red shifted compared to its Brooker metamer [8]. This comparative analysis clearly shows the impact of metamerism NLO properties. Also, it was pointed out that Reichardt’s type is definitely a better chromophore (NLO-phore) than the Brooker type of zwitterion, with the tetrazine as the bridge. Comparing the results from various methodologies (Table 6 and Table S6 – SI Text), it was observed that while B3LYP predicted the lowest value, at the same time, ωB97xD predicted the highest value of hyperpolarizability (comparable to HF and CAM-B3LYP values).

#### Five-membered heteroaromatics (single heteroatom) as bridges

In the cases of five-membered aromatic rings as bridges, three well-known heteroaromatic five-membered rings were investigated. With pyrrole, furan, and thiophene as the bridges, we were able to get Molecules 11, 12, and 13 respectively. Optimized geometric conformations and various computed molecular properties for these three molecules are shown in Table 7 and Table S7 (ωB97xD and CAM-B3LYP data in Table 7 and HF and B3LYP data in Table S7 – SI Text). While Molecule 12 showed almost planar conformation (D-side: − 0.4° and A-side: − 2.4°), at the same time, Molecules 11 and 13 showed twisted conformations (Molecule 11: D-side: + 14.8° and A-side: + 19.5° and Molecule 13: D-side: + 3.4° and A-side: − 19.9°). All three zwitterions showed reasonably good dipole moments and were found to be intermediate between Molecules 1 and 2 (indicates the roles played by these bridges). Except Molecule 12, other two molecules showed only one contributing tensor (*x*-component) to their total ground state dipole moments (Molecule 12 showed some minor contribution from the *µ*_*y*_ tensor). For Molecules 11–13, enhanced polarizabilities (*α*) were observed compared to Molecule 1, and at the same time, very minor differences were observed compared to Molecule 2. Also, for all the three molecules, enhanced hyperpolarizabilities (*β*) were observed compared to Molecule 1, and reduced values of *β* were observed compared to Molecule 2.

**Table 7:** Optimized geometries, ground state dipole moments (*μ*_*g*_) and ground to excited state dipole differences (*Δμ*_*ge*_) in Debye, excitation energies (*ΔE*_*ge*_) in eV, absorption maxima (*λ*_*max*_) in nm, average polarizabilities (*<α>***)** in 10^-24^ esu**,** first hyperpolarizabilities (*β*_*0*_) in 10^-30^ esu oscillator strengths *f*_*ge*_ (unitless), coefficients of maximum contributing transition *C*_*i*_ (unitless) with frontier molecular orbitals, of Molecules 11, 12, & 13, computed using CAM-B3LYP and ωB97xD methodologies

**Molecule #**	**Methodologies**	***μ***_***g***_	***<α>***	***β***_***0***_	***Δμ***_***ge***_	***ΔE***_***ge***_	***λ***_***max***_	***f***_***ge***_	***C***_***i***_
**Molecule 11**	**CAM-B3LYP**	25.5	67.7	443.7	38.0	1.7	707.0	1.04	0.71
	**ωB97xD**	27.2	66.7	615.1	39.6	1.8	702.8	1.03	0.70
	HOMO				**→**	LUMO			
**Molecule 12**	**CAM-B3LYP**	24.0	65.4	339.6	36.6	1.8	673.9	1.12	0.71
	**ωB97xD**	25.2	65.3	450.8	37.9	1.8	669.5	1.13	0.70
	HOMO				**→**	LUMO			
**Molecule 13**	**CAM-B3LYP**	24.7	72.5	363.1	38.5	1.8	667.6	1.35	0.71
	**ωB97xD**	26.3	72.8	524.5	40.0	1.8	675.5	1.31	0.69
	HOMO				**→**	LUMO			

Now, when Molecules 11–13 were compared with their respective Brooker metamers (already reported in the literature [8]), no significant differences with the dipole moments and polarizabilities were observed. But at the same time, hyperpolarizabilities (*β*) values were found to be showing some peculiar behaviors (Molecule 11: 615.1 × 10^−30^ esu compared to its metamer 349.6 × 10^−30^ esu, Molecule 12: 450.8 × 10^−30^ esu compared to its metamer 746.3 × 10^–30^ esu, and Molecule 13: 524.5 × 10^−30^ esu compared to its metamer 215.9 × 10^−30^ esu). It is interesting to note that while in the case of pyrrole (Molecule 11) and thiophene (Molecule 13), enhanced hyperpolarizabilities were observed for the Reichardt types compared to Brooker’s types, at the same time, a completely reverse impact was observed (enhanced *β* for Brooker’s than Reichardt’s) in the case of Molecule 12 (furan bridge). Analysis of the electronic absorptions indicates similar characteristics for Molecules 11–13. Like all the earlier cases, here, also the excitations were found to be of HOMO to LUMO transitions. Natures of these two orbitals in all the three molecules were found to be delocalized (with partially localized electron densities at the D-sides in HOMOs and at the A-sides in LUMOs). Now, about the *λ*_max_ values, red-shifted absorptions for all the three molecules compared to Molecule 1 and slightly blue-shifted absorptions compared to Molecule 2 were observed. Now, comparing Molecules 11–13 with their corresponding Brooker type metamers [8], all of them showed slightly red-shifted absorption maxima (*λ*_max_ values, for Molecule 11: 702.8 nm compared to 566.0 nm, Molecule 12: 669.5 nm compared to 562.2 nm, and Molecule 13: 675.5 nm compared to 573.3 nm).

Analysis of the results from all the four methodologies for Molecules 11–13 (Table 7 and Table S7 – SI Text) indicates very similar trends to that of the previously discussed molecules. Now, about the quantitative values, for the dipole moments, HF predicted the largest values and B3LYP predicted the lowest values. About the polarizabilities, HF predicted lower values and all the three DFT methodologies predicted relatively larger values (found to be in the similar range). Now, about the hyperpolarizabilities, while Molecule 11 showed the largest value with ωB97xD, at the same time, for Molecules 12 and 13, HF predicted the largest values (slightly larger than ωB97xD). For all the three cases, B3LYP showed the lowest values of hyperpolarizabilities. For the analysis of the absorption maxima for all the three cases, while HF predicted the relatively lower values, at the same time, all the DFT methodologies predicted very similar absorption maxima.

#### Five-membered heteroaromatics (double heteroatoms) as the bridges

With the double heteroatoms with five-membered aromatic rings, imidazole, oxazole, and thiazole moieties as bridges, we were able to get two different molecules in each bridge case, based on the positioning of the two heteroatoms present in these aromatic bridges (either closer towards D- or towards A-sides). Obtained six Reichardt’s types of zwitterions were Molecules 14a and 14b (imidazole as the bridge), Molecules 15a and 15b (oxazole as the bridge), and Molecules 16a and 16b (thiazole as the bridge). Optimized geometric conformation and various computed molecular properties of all these six molecules are shown in Table 8 and Table S8 (ωB97xD and CAM-B3LYP data in Table 8 and HF and B3LYP data in Table S8 – SI Text). While Molecules 15a, 15b, and 16b all showed nearly planar conformations, at the same time, Molecules 14a, 14b, and 16a showed twisted conformations. Twisted conformations for Molecules 14a and 14b may be due to the presence of the N–H linkage (capable of exerting some short of steric interactions with the adjacent rings), for Molecule 14a, inter-ring junction twisting of + 7.8° (D-side) and − 16.6° (A-side), and for Molecule 14b, inter-ring junction twisting of + 10.9° (D-side) and − 11.0° (A-side). For Molecule 16a, only one-side twisted conformation (A-side: 22.0°) was observed (H…H interaction present in the A-side might be efficiently exerting steric repulsion). All the six molecules showed zwitterionic characters, with reasonably large values dipole moments with only one contributing tensor (*x*-component) to the total ground state dipole moments, and quantitative values were between Molecules 1 and 2. Like the dipole moments, for Molecules 14(a,b)–16(a,b), enhanced polarizabilities (*α*) were observed compared to Molecule 1, but compared to Molecule 2, nearly similar values of polarizabilities were observed. With respect to trends in hyperpolarizabilities, all the six molecules showed enhanced values of *β* compared to Molecule 1 and at the same time reduced values of *β* compared to Molecule 2.

**Table 8:** Optimized geometries, ground state dipole moments (*μ*_*g*_) and ground to excited state dipole differences (*Δμ*_*ge*_) in Debye, excitation energies (*ΔE*_*ge*_) in eV, absorption maxima (*λ*_*max*_) in nm, average polarizabilities (*<α>***)** in 10^-24^ esu**,** 1^ST^ hyperpolarizabilities (*β*_*0*_) in 10^-30^ esu, oscillator strengths *f*_*ge*_ (unitless), coefficients of maximum contributing transition *C*_*i*_ (unitless) with frontier molecular orbitals, of Molecules 14a, 14b, 15a, 15b, 16a and 16b, computed using CAM-B3LYP and ωB97xD methodologies

**Molecule #**	**Methodologies**	***μ***_***g***_	***<α>***	***β***_***0***_	***Δμ***_***ge***_	***ΔE***_***ge***_	***λ***_***max***_	***f***_***ge***_	***C***_***i***_
**Molecule 14a**	**CAM-B3LYP**	26.6	67.1	499.9	36.7	1.7	732.1	0.94	0.71
	**ωB97xD**	28.1	65.3	659.2	40.1	1.7	715.6	0.94	0.70
	HOMO				**→**	LUMO			
**Molecule 14b**	**CAM-B3LYP**	25.0	69.1	498.8	37.5	1.7	748.6	0.97	0.72
	**ωB97xD**	26.8	67.9	709.1	39.1	1.7	743.9	0.95	0.70
	HOMO				**→**	LUMO			
**Molecule 15a**	**CAM-B3LYP**	25.0	63.9	411.3	13.0	1.8	693.8	0.98	0.71
	**ωB97xD**	26.3	63.0	516.4	38.4	1.8	678.4	1.01	0.70
	HOMO				**→**	LUMO			
**Molecule 15b**	**CAM-B3LYP**	23.5	67.0	394.7	11.0	1.7	725.0	1.00	0.72
	**ωB97xD**	24.9	67.0	544.2	12.8	1.7	718.6	1.02	0.70
	HOMO				**→**	LUMO			
**Molecule 16a**	**CAM-B3LYP**	25.4	69.0	411.6	38.4	1.9	664.7	1.21	0.70
	**ωB97xD**	27.0	68.2	545.4	39.9	1.8	664.8	1.18	0.68
	HOMO				**→**	LUMO			
**Molecule 16b**	**CAM-B3LYP**	23.9	73.0	397.7	10.1	1.8	692.3	1.28	0.71
	**ωB97xD**	25.3	73.5	552.1	11.5	1.8	693.0	1.30	0.69
	HOMO				**→**	LUMO			

Then, these six Reichardt types of zwitterions were compared with their respect Brooker metamers [8]. Though no significant differences were observed with the dipole moments and polarizabilities in respective metameric pairs, but hyperpolarizability (*β*) values were found to be different from each other. The observed *β* values were as follows: Molecule 14a: 659.2 × 10^−30^ esu (metamer 427.0 × 10^−30^ esu), Molecule 14b: 709.1 × 10^−30^ esu (metamer 419.3 × 10^−30^ esu), Molecule 15a: 516.4 × 10^−30^ esu (metamer 310.7 × 10^−30^ esu), Molecule 15b: 544.2 × 10^−30^ esu (metamer 282.4 × 10^−30^ esu), Molecule 16a: 545.4 × 10^−30^ esu (metamer 248.8 × 10^−30^ esu), and Molecule 16b: 552.1 × 10^–30^ esu (metamer 227.8 × 10^−30^ esu). For all the cases, enhanced hyperpolarizabilities were observed for the Reichardt types compared to Brooker types. Among the bridges, imidazole bridged molecules were found to be more efficient compared to the other two bridge cases. For the analysis of the electronic absorptions for all the six molecules, while all of them showed reasonably strong absorption oscillator strengths (*f*_ge_), at the same time, except Molecules 15b and 16b, all the other molecules showed large values of Δ*μ*_ge_. Like all the earlier cases, here, also the excitations were found to be of HOMO to LUMO transitions. Natures of these two participating orbitals were found to be delocalized (with partially localized electron densities at the D-sides in HOMOs and at the A-sides in LUMOs) in all the six molecules. Analysis of the *λ*_max_ values for all the six molecules indicated red-shifted absorptions compared to Molecule 1 and slightly blue-shifted absorptions compared to Molecule 2. Now, comparing all these six molecules with their respective Brooker metamers [8], all showed slightly red-shifted absorption maxima (*λ*_max_ values, for Molecule 14a: 715.6 nm compared to 585.9 nm, Molecule 14b: 743.9 nm compared to 597.9 nm, Molecule 15a: 678.4 nm compared to 568.6 nm, Molecule 15b: 718.6 nm compared to 595.4 nm, Molecule 16a: 664.8 nm compared to 563.8 nm, and Molecule 16b: 693.0 nm compared to 601.4 nm).

Analysis of the results from all the four methodologies, for the Molecules 14(a,b)–16(a,b) (Table 7 and Table S7 – SI Text), showed the observed trends to be very similar to those of the previously discussed other molecules. Quantitatively, while HF predicted the largest values and B3LYP predicted the lowest values of the dipole moments, at the same time, in the cases of polarizabilities, HF predicted low values and all the three DFT methodologies predicted relatively larger values (almost similar range). For the analysis of the hyperpolarizability values, different trends were observed. For Molecules 14a, 14b, and 16a, *β* values were found to be largest in ωB97xD and lowest in B3LYP methodologies (these three showed twisted conformations). At the same time, Molecule 15b and 16b showed the largest values of *β* for HF and the lowest values of *β* for B3LYP methodologies. Although Molecule 15a showed a similar trend like 15b and 16b, but the observed *β* value of HF was found to be only slightly larger than the ωB97xD value. For the analysis of the absorption maxima (*λ*_max_) for all the six molecules, it was observed that in general, HF predicted the relatively lower values; at the same time, all the DFT methodologies predicted very similar absorption maxima (and larger values than HF).

### Comparison with traditional bridges

Then, structure–property correlations for Reichardt’s type of zwitterions, with some well-known nonaromatic bridges, were studied in numerous earlier works related to NLO. Details about the natures of these types of bridges can be found in a relatively recent review article by Liu et al. [19] Investigated Reichardt’s type zwitterions were with a *π*-bridges or -CH = CH- or -C≡C- (Molecules 17 and 18 respectively), a nonconjugated *σ*-bridge or -CH_2_-CH_2_- (Molecule 19), and cumulenic bridges (Molecule 20: -CH = C = CH- or the allenic linkage as the bridge and Molecule 21: -CH = C = C = CH- or the cumulenic linkage as the bridge). Optimized geometric conformation and various computed molecular properties of these six Reichardt types of zwitterions with the well-known traditional bridges are shown in Table 9 and Table S9 (ωB97xD and CAM-B3LYP data in Table 9 and HF and B3LYP data in Table S9 – SI Text).

**Table 9:** Optimized geometries, ground state dipole moments (*μ*_*g*_) and ground to excited state dipole differences (*Δμ*_*ge*_) in Debye, excitation energies (*ΔE*_*ge*_) in eV, absorption maxima (*λ*_*max*_) in nm, average polarizabilities (*<α>***)** in 10^-24^ esu**,** 1^ST^ hyperpolarizabilities (*β*_*0*_) in 10^-30^ esu, oscillator strengths *f*_*ge*_ (unitless), coefficients of maximum contributing transition *C*_*i*_ (unitless) with frontier molecular orbitals, of Molecules 17-21, computed using CAM-B3LYP and ωB97xD methodologies

**Molecule #**	**Methodologies**	***μ***_***g***_	***<α>***	***β***_***0***_	***Δμ***_***ge***_	***ΔE***_***ge***_	***λ***_***max***_	***f***_***ge***_	***C***_***i***_
**Molecule 17**	**CAM-B3LYP**	21.4	55.0	206.0	34.5	2.2	560.5	1.44	0.70
	**ωB97xD**	22.3	55.1	258.7	35.4	2.2	562.0	1.43	0.69
	HOMO				**→**	LUMO			
**Molecule 18**	**CAM-B3LYP**	22.7	58.4	287.2	9.50	2.1	589.4	1.39	0.71
	**ωB97xD**	23.7	58.3	366.8	37.0	2.1	585.2	1.41	0.70
	HOMO				**→**	LUMO			
**Molecule 19**	**CAM-B3LYP**	30.9	62.7	926.3	39.7	0.9	1349.8	0.27	0.73
	**ωB97xD**	33.0	53.2	1699.6	25.4	0.95	1295.1	0.21	0.70
	HOMO				**→**	LUMO			
**Molecule 20**	**CAM-B3LYP**	26.9	39.8	27.7	28.2	3.0	410.3	1.10	0.68
	**ωB97xD**	25.3	38.8	151.7	23.3	1.3	937.9	0.02	0.67
	HOMO				**→**	LUMO			
**Molecule 21**	**CAM-B3LYP**	21.0	67.1	80.4	36.0	2.2	559.6	1.90	0.69
	**ωB97xD**	21.3	67.8	82.2	36.4	2.2	563.3	1.88	0.68
	HOMO				**→**	LUMO			

#### π-Conjugated bridges

Numerous studies can be found in the literature related to D-*π*-A types of push–pull organic NLO-phores (review articles: references [52, 53, 57, 97]). While Molecule 18 adopted fully planar conformation (linear arrangements), at the same time, Molecule 17 showed a twisted conformation (A-side: − 21.0° and D-side: − 7.0°). For Molecules 17 and 18, slightly enhanced dipole moments were observed compared to Molecule 1 and slightly reduced values compared to Molecule 2. Now, about the polarizabilities, both for Molecule 17 and 18, intermediated values between Molecule 1 (lower) and Molecule 2 (larger) were observed. Similarly, though intermediate values (between Molecules 1 and 2) of first hyperpolarizabilities were observed for Molecules 17 and 18, but quantitatively, they were found to be much lower than Molecule 2. Compared to Molecule 2, both the molecules showed blue-shifted absorption maxima, with strong absorption oscillator strengths, and HOMO to LUMO transitions (orbitals were found to be delocalized in natures) during the excitation processes. All these observations clearly signify the importance of aromatic bridges over the traditional *π*-bridges.

#### Nonconjugated σ-bridge

Some earlier works investigated nonconjugated *σ*-bridges for NLO-phore designs and demonstrated that in such kinds of molecules, the dominating charge transfer effects were TBI (through bond interaction) types [35, 60, 61]. Optimized conformation of Molecule 19 showed a step-like all trans-configuration of the D and A fragments, arranged in anti-co-facial arrangement around the *σ*-bridge [35]. Dipole moment of Molecule 19 was found to be like that of Molecule 2, but much larger than Molecule 1. Now, for the polarizability of Molecule 19, it was found to be larger than Molecule 1, but lower than Molecule 2. At the same time, Molecule 19 showed larger first hyperpolarizability (*β*_0_ = 1699.6 × 10^−30^ esu) compared to Molecule 2 (*β*_0_ = 1291.2 × 10^−30^ esu). Compared to Molecule 2, Molecule 19 showed red-shifted absorption maximum (*λ*_max_ = 1295.1 nm), but with a relatively weaker absorption oscillator strengths and HOMO to LUMO transitions (orbitals were localized in natures: HOMO in the D-side and LUMO in the A-side) during the excitation processes. With the comparable molecular properties shown by most of the aromatic bridges with respect to the *σ*-bridge, one major advantage can be seen that, with the aromatic bridges, they are associated with strong charge transfer characteristics with much stronger oscillator strengths, than the *σ*-bridged molecules.

#### Cumulenic bridges

Few earlier studies report the roles of cumulenic bridges on NLO, and advantages of mutually perpendicular *π*-orbitals present in these cumulene bridges were also discussed for functional molecular materials [36, 98]. While Molecule 21 (with odd number of cumulenic double bonds or even number of cumulenic C-atoms) adopted fully planar conformation (D and A in trans-like conformation), at the same time, Molecule 20 showed a twisted conformation (intermediate conformation between the *σ*-bridge and *π*-bridge cases). For Molecules 20 and 21, slightly enhanced dipole moments were observed compared to Molecule 1 and reduced values compared to Molecule 2. But, for polarizabilities, while Molecule 20 showed much smaller value than Molecule 2 (like Molecule 1), at the same time, Molecule 21 showed larger value compared to Molecule 1, but lower value compared to Molecule 2. Now, about the hyperpolarizabilities, both showed very low values, even lowered compared to Molecule 1 (Molecule 20: *β*_0_ = 151.7 × 10^−30^ esu Molecule 21: *β*_*0*_ = 82.2 × 10^−30^ esu, and Molecule 1: *β*_*0*_ = 215.0 × 10^−30^ esu). Compared to Molecule 2, while Molecule 20 showed red-shifted absorption maximum (with a very weak absorption oscillator strength), at the same time, a stronger, blue-shifted absorption was observed for Molecule 21. Both the molecules showed HOMO to LUMO transitions during the excitations. About these orbitals, localized and delocalized orbital electron densities were observed for Molecules 20 and 21 respectively. Though results from cumulenic bridges clearly highlight the importance of aromatic bridges, at the same time, it also shows that cumulenic bridged Reichardt’s types of zwitterions may not be very suitable as NLO-phores.

## Conclusions

This work reports a systematic computational study (with HF, B3LYP, CAM-B3LYP, and ωB97xD methodologies) to account for the roles of a wide range of aromatic and nonaromatic bridges, on various response properties of 27 Reichardt’s types of zwitterions. The primary intension of this study id to see the effects of metamerism (Reichardt’s vs Brooker’s) on NLO responses. When compared with their corresponding Brooker types of zwitterions, in general, enhanced response properties were observed (mostly in the range of 2–3 times enhanced). This clearly shows the impacts of metamerism on the structure–property correlations, and they were highlighted in this report. It has been highlighted that, like the Brooker types, Reichardt’s types of zwitterions can also be considered suitable materials for nonlinear optical applications, and at the same time, Reichardt’s types are more efficient. Investigated Reichardt’s types of zwitterions showed unidirectional response properties (*μ* and *β*). Thus, they have been suggested as suitable 1D or pseudo-1D functional molecular materials, and their possible applications for the field of NLO as well as other areas of technological interests are also suggested. In general, enhanced values of dipole moments and hyperpolarizabilities were observed for these aromatically bridged Reichardt types of zwitterions, compared to the Reichardt type of D-A directly connected (no bridge case) zwitterion (mostly in the range of 6–7 times enhancements were observed). Based on the structure–property analysis presented in this work, one can say that aromatic bridges in these Reichardt types of zwitterions are capable of efficiently influencing the charge transfer characteristics and thus strongly affecting their linear as well as nonlinear optical response properties. From the chromophore design point of view, with the possibilities of so many varieties of aromatic rings, one can be able to produce a larger array of chromophores (both Reichardt’s: studied in this work, and Brooker’s types: studied in earlier works) with many interesting characteristics. Thus, this work suggests that the aromaticity control over the bridge in D-bridge-A types of chromophores is definitely an efficient and effective chromophore design strategy and can be easily adopted by researchers working in the areas of functional molecular materials.

### Supplementary information

Below is the link to the electronic supplementary material.

Supplementary file1 (DOCX 84 KB)

## Data Availability

Optimized (ωB97xD) geometries of all the molecules in the form of Cartesian coordinates and, also, computed molecular electronic properties of all the molecules from HF and B3LYP methodologies (Tables S1–S9) are provided as supporting information data. Optimized coordinates for other methodologies can be obtained from the author, through email request. A concise description of CPHF theory used in the computations of response properties is also provided.
