# Supplementary material for: Enhanced molecular first hyperpolarizabilities with Reichardt’s type of zwitterions: a computational study on roles of various monocyclic aromatic bridges
Source: J Mol Model. 2024 Jul 26;30(8):284. doi: 10.1007/s00894-024-06055-3 (PMC11282158; doi:10.1007/s00894-024-06055-3)
Supplement: Supplementary file 1 — Supplementary file1 (DOCX 84 KB) [file 894_2024_6055_MOESM1_ESM.docx]

**Enhanced Molecular First Hyperpolarizabilities with Reichardt’s Type of Zwitterions: A Computational Study on Roles of Various Monocyclic Aromatic Bridges**

**Divya Pant**, **Sanyasi Sitha***

Department of Chemical Sciences, Auckland Park Kingsway (APK) Campus, University of Johannesburg, PO Box 524, Auckland Park, 2006, Johannesburg, South Africa.

**--------------------------------------------------------------------------------------------------**

**Coupled-Perturbed Theory:**

Coupled-perturbed theory**^1,2^** used in this report for the computations of the tensorial components of hyperpolarizabilities, is built on the principles of response theory, where time-dependent perturbation theory is generally applied to the system under investigation. With the application of perturbation with an external electrostatic potential, then with the inclusion of the perturbative field in the Hamiltonian, the new Hamiltonian can be represented as (Eq. 1),

$\hat{H}(F)= \hat{H_{0}}+ \hat{V}$ **Eq. 1**

Where, $\hat{H} \left( F \right)$is the perturbed Hamiltonian in presence of the applied field, $\hat{H}$_0_ is the unperturbed zero-field molecular Hamiltonian, and $\hat{V}$is the perturbation potential (usually very small). Then with the normalized wave functions, field dependent energy, *E(F)* of the system can be represented as in Eq. 2.

$E\left( F \right)=\left\langle\left| \hat{} \right|\hat{H}(F)\left| \hat{} \right| \right\rangle$ **Eq. 2**

Similarly, field-dependent dipole moment, *μ(F)* of the system can be assessed using Eq. 3.

$\mu\left( F \right)=\left\langle\left| \hat{} \right|q_{i}\left( F \right)r_{i}(F)\left| \hat{} \right| \right\rangle$ **Eq. 3**

From the well-known Maxwell’s equations, using the general approximation, like Taylor power series expansion, the induced dipole moment when the system is interacting with an external perturbative electric field, can be represented as in Eq. 4.

**Eq. 4**

$$\mu_{i}\left( F \right)= \mu_{i}^{0}+ \sum_{j} \alpha_{ij}F_{j} + \frac{1}{2!}\sum_{j<k} \beta_{ijk}F_{j}F_{k} + \frac{1}{3!}\sum_{j<k<l} \gamma_{ijkl}F_{j}F_{k}F_{l}+\ldots$$

Where, $\mu_{i}^{0}$ is the intrinsic dipole moment of the molecule (zero field). Here the polarizabilities and hyperpolarizabilities are usually found by differentiating $\mu_{i}\left( F \right)$ with respect to the perturbative field (Eq. 5a-5c).

${\alpha_{iJ}= \left. \frac{\mu_{i}}{F_{j}} \right|}_{F=0}$ **Eq. 5a**

${\beta_{iJk}= \left. \frac{{}^{\boldsymbol{2}}\mu_{i}}{F_{j}F_{k}} \right|}_{F=0}$ **Eq. 5b**

${\gamma_{iJkl}= \left. \frac{{}^{\boldsymbol{3}}\mu_{i}}{F_{j}F_{k}F_{l}} \right|}_{F=0}$ **Eq. 5c**

Instead of the dipole derivatives, similar expressions for α, β, and γ, can be obtained with the molecular energy, as molecular dipole moment can be represented as the negative field derivative of the energy (Eq. 6).

${\mu_{iJ}=- \left. \frac{E}{F_{i}} \right|}_{F=0}$ **Eq. 6**

Relationships shown above for the α, β, and γ (Eq. 5a-5c or equivalent expressions using Eq. 4 and Eq. 6) are applicable only for the static field limits and these derivatives are generally compute using either numerical differentiations or analytic gradient techniques. In the coupled-perturbed technique, the latter approach is used to compute NLO responses and other related properties.

**References:**

1. Pople JA, McIven JM, Ostlund NS (1968) Self‐Consistent Perturbation Theory. I. Finite Perturbation Methods. J Chem Phys 49: 2960.
2. Hush NS, Williams ML (1970) Finite-perturbation SCF valence-shell calculations of molecular polarizability and hyperpolarizability components. Chem Phys Lett 5: 507.

**--------------------------------------------------------------------------------------------------**

**Section-S1: The spectroscopic data for all compounds was obtained by TDDFT vertical excitation investigations, employing the HF, B3LYP, and CAM-B3LYP techniques. Please consult the main text of the article for the visualization of molecular structures and their corresponding numbering for the respective compounds.**

Ground state dipole moments (**µ*_g_***) and ground to excited state dipole differences (**Δµ*_ge_***) in Debye, excitation energies (**ΔE*_ge_***) in eV, absorption maxima (**λ*_max_***) in nm, average polarizabilities (**<α>)** in 10^-24^ esu**,** 1^ST^ hyperpolarizabilities (**β_0_**) in 10^-30^ esu, oscillator strengths ***f_ge_*** (unitless), coefficients of maximum contributing transitions **C*_i_*** (unitless). Here, the frontier molecular orbitals, HOMO represents Highest Occupied Molecular Orbital and LUMO represents the Lowest Unoccupied Molecule Orbital. Tables S1 – S9 show the spectroscopic data for all the molecules computed using the above three methodologies.

**Table S1**

| **Molecule** | **Methods** | **µ_g_** | **<α>** | **β_0_** | **Δµ_ge_** | **ΔE_ge_** | **λ_max_** | ***f_ge_*** | Orbitals involved in Excitations | | | **C_i_** |
| --- | --- | --- | --- | --- | --- | --- | --- | --- | --- | --- | --- | --- |
| **1** | HF | 24.6 | 27.1 | 120.5 | 30.9 | 3.0 | 410.8 | 0.45 | HOMO | **→** | LUMO | 0.67 |
|  | B3LYP | 17.0 | 39.0 | 88.9 | 7.8 | 2.1 | 590.0 | 0.67 |  |  |  | 0.73 |

**Table S2**

| **Molecule** | **Methods** | **µ_g_** | **<α>** | **β_0_** | **Δµ_ge_** | **ΔE_ge_** | **λ_max_** | ***f_ge_*** | Orbitals involved in Excitations | | | **C_i_** |
| --- | --- | --- | --- | --- | --- | --- | --- | --- | --- | --- | --- | --- |
| **2** | HF | 41.3 | 43.1 | 377.2 | 48.3 | 2.6 | 485.0 | 0.48 | HOMO | **→** | LUMO | 0.66 |
|  | B3LYP | 25.0 | 79.4 | 208.4 | 12.3 | 1.5 | 804.2 | 0.95 |  |  |  | 0.76 |

**Table S3**

| **Molecules** | **Methods** | **µ_g_** | **<α>** | **β_0_** | **Δµ_ge_** | **ΔE_ge_** | **λ_max_** | ***f_ge_*** | Orbitals involved in Excitations | | | **C_i_** |
| --- | --- | --- | --- | --- | --- | --- | --- | --- | --- | --- | --- | --- |
| **3a** | HF | 38.9 | 45.7 | 556.9 | 48.4 | 2.5 | 490.9 | 0.86 | HOMO | **→** | LUMO | 0.65 |
|  | B3LYP | 23.6 | 77.2 | 217.6 | 10.6 | 1.6 | 757.3 | 1.06 |  |  |  | 0.76 |
| **3b** | HF | 38.9 | 46.7 | 415.3 | 47.8 | 2.6 | 476.2 | 0.76 | HOMO | **→** | LUMO | 0.64 |
|  | B3LYP | 24.9 | 77.1 | 207.5 | 37.3 | 1.6 | 777.3 | 0.94 |  |  |  | 0.75 |

**Table S4**

| **Molecules** | **Methods** | **µ_g_** | **<α>** | **β_0_** | **Δµ_ge_** | **ΔE_ge_** | **λ_max_** | ***f_ge_*** | Orbitals involved in Excitations | | | **C_i_** |
| --- | --- | --- | --- | --- | --- | --- | --- | --- | --- | --- | --- | --- |
| **4** | HF | 35.4 | 51.0 | 549.8 | 47.3 | 2.6 | 475.2 | 1.40 | HOMO | **→** | LUMO | 0.62 |
|  | B3LYP | 23.1 | 74.6 | 214.2 | 36.1 | 1.7 | 714.8 | 1.11 |  |  |  | 0.74 |
| **5** | HF | 37.0 | 48.4 | 530.6 | 47.6 | 2.6 | 477.8 | 1.11 | HOMO | **→** | LUMO | 0.64 |
|  | B3LYP | 23.7 | 75.4 | 212.3 | 35.3 | 1.6 | 771.9 | 0.83 |  |  |  | 0.71 |
| **6a** | HF | 37.3 | 46.1 | 653.4 | 47.3 | 2.5 | 502.7 | 0.94 | HOMO | **→** | LUMO | 0.65 |
|  | B3LYP | 22.5 | 75.3 | 234.6 | 35.7 | 1.7 | 742.9 | 1.09 |  |  |  | 0.75 |
| **6b** | HF | 38.1 | 46.3 | 351.0 | 29.3 | 2.7 | 460.4 | 0.79 | HOMO | **→** | LUMO | 0.64 |
|  | B3LYP | 24.8 | 75.6 | 210.0 | 37.1 | 1.6 | 769.3 | 0.92 |  |  |  | 0.75 |

*For Molecule 5, in all the three methodologies, minor contributions were observed from the y-components to the total dipole moment, in respective methodologies (HF: µ**_y_ =** 3.59 D and B3LYP: µ**_y_ =** 3.27 D).

**Table S5**

| **Molecules** | **Methods** | **µ_g_** | **<α>** | **β_0_** | **Δµ_ge_** | **ΔE_ge_** | **λ_max_** | ***f_ge_*** | Orbitals involved in Excitations | | | **C_i_** |
| --- | --- | --- | --- | --- | --- | --- | --- | --- | --- | --- | --- | --- |
| **7a** | HF | 34.2 | 50.5 | 601.9 | 46.2 | 2.6 | 481.0 | 1.41 | HOMO | **→** | LUMO | 0.63 |
|  | B3LYP | 22.3 | 73.0 | 236.1 | 29.7 | 1.6 | 774.5 | 0.34 |  |  |  | 0.49 |
| **7b** | HF | 34.9 | 50.0 | 469.5 | 46.7 | 2.7 | 462.0 | 1.41 | HOMO | **→** | LUMO | 0.62 |
|  | B3LYP | 23.2 | 73.5 | 209.1 | 32.9 | 1.7 | 746.9 | 0.58 |  |  |  | 0.59 |
| **8** | HF | 29.3 | 39.6 | 107.7 | 24.8 | 3.1 | 399.2 | 0.26 | HOMO | **→** | LUMO | 0.65 |
|  | B3LYP^*^ | 23.6 | 67.0 | 438.6 | 18.0 | 0.6 | 2093.0 | 0.06 |  |  |  | 0.74 |
| **9** | HF | 31.3 | 41.9 | 152.4 | 26.3 | 2.8 | 435.0 | 0.30 | HOMO | **→** | LUMO | 0.65 |
|  | B3LYP^*^ | 23.3 | 70.8 | 285.0 | 16.7 | 0.7 | 1640.8 | 0.12 |  |  |  | 0.75 |

*For Molecule 8, the absorption was found to be in the far-IR region in B3LYP and near-IR region in CAM-B3LYP methodologies, with weaker oscillator strengths in both the cases.

For Molecules 9, in all the three methodologies, minor contributions from the y-components to the total dipole moments were observed (HF: µ**_y_ =** 5.58 D and B3LYP: µ**_y_ =** 5.16 D).

Now adiabatic absorption for Molecule 9, near-IR absorption in B3LYP and slightly far-IR absorption in CAM-B3LYP methodologies were observed. Also, in both the methodologies, relatively lower values of oscillator strengths were observed.

**Table S6**

| **Molecules** | **Methods** | **µ_g_** | **<α>** | **β_0_** | **Δµ_ge_** | **ΔE_ge_** | **λ_max_** | ***f_ge_*** | Orbitals involved in Excitations | | | **C_i_** |
| --- | --- | --- | --- | --- | --- | --- | --- | --- | --- | --- | --- | --- |
| **10** | HF | 32.1 | 52.9 | 554.3 | 45.2 | 2.6 | 472.5 | 1.72 | HOMO | **→** | LUMO | 0.63 |
|  | B3LYP | 21.9 | 71.5 | 225.3 | 21.9 | 1.7 | 703.0 | 1.12 |  |  |  | 0.74 |

**Table S7**

| **Molecules** | **Method** | **µ_g_** | **<α>** | **β_0_** | **Δµ_ge_** | **ΔE_ge_** | **λ_max_** | ***f_ge_*** | Orbitals involved in Excitations | | | **C_i_** |
| --- | --- | --- | --- | --- | --- | --- | --- | --- | --- | --- | --- | --- |
| **11** | HF | 36.1 | 43.5 | 530.0 | 44.7 | 2.3 | 537.2 | 0.64 | HOMO | **→** | LUMO | 0.65 |
|  | B3LYP | 21.9 | 67.1 | 164.3 | 33.6 | 1.7 | 728.4 | 0.88 |  |  |  | 0.74 |
| **12** | HF | 32.7 | 50.4 | 699.0 | 43.5 | 2.2 | 563.5 | 0.98 | HOMO | **→** | LUMO | 0.65 |
|  | B3LYP | 21.0 | 64.6 | 141.5 | 32.6 | 1.8 | 694.9 | 0.93 |  |  |  | 0.74 |
| **13** | HF | 36.8 | 47.8 | 544.9 | 46.1 | 2.4 | 523.7 | 0.78 | HOMO | **→** | LUMO | 0.64 |
|  | B3LYP | 21.5 | 71.0 | 151.0 | 34.4 | 1.8 | 681.0 | 1.14 |  |  |  | 0.74 |

**Table S8**

| **Molecules** | **Methods** | **µ_g_** | **<α>** | **β_0_** | **Δµ_ge_** | **ΔE_ge_** | **λ_max_** | ***f_ge_*** | Orbitals involved in Excitations | | | **C_i_** |
| --- | --- | --- | --- | --- | --- | --- | --- | --- | --- | --- | --- | --- |
| **14a** | HF | 36.4 | 41.7 | 428.1 | 44.5 | 2.4 | 511.1 | 0.60 | HOMO | **→** | LUMO | 0.65 |
|  | B3LYP | 22.6 | 67.5 | 174.2 | 34.0 | 1.6 | 765.3 | 0.80 |  |  |  | 0.74 |
| **14b** | HF | 35.8 | 42.4 | 584.0 | 44.0 | 2.2 | 557.2 | 0.57 | HOMO | **→** | LUMO | 0.67 |
|  | B3LYP | 20.9 | 67.9 | 161.6 | 32.6 | 1.6 | 761.4 | 0.84 |  |  |  | 0.75 |
| **15a** | HF | 33.4 | 45.5 | 523.8 | 43.3 | 2.4 | 519.2 | 0.89 | HOMO | **→** | LUMO | 0.65 |
|  | B3LYP | 21.6 | 64.6 | 165.5 | 10.3 | 1.7 | 733.3 | 0.81 |  |  |  | 0.74 |
| **15b** | HF | 33.4 | 46.4 | 716.4 | 42.9 | 2.1 | 581.9 | 0.73 | HOMO | **→** | LUMO | 0.66 |
|  | B3LYP | 19.7 | 65.6 | 137.8 | 8.01 | 1.6 | 733.3 | 0.88 |  |  |  | 0.75 |
| **16a** | HF | 35.5 | 45.2 | 336.3 | 44.1 | 2.6 | 478.4 | 0.72 | HOMO | **→** | LUMO | 0.62 |
|  | B3LYP | 22.1 | 69.9 | 181.8 | 34.2 | 1.8 | 704.3 | 0.98 |  |  |  | 0.73 |
| **16b** | HF | 34.0 | 55.9 | 915.6 | 45.9 | 2.1 | 577.0 | 1.15 | HOMO | **→** | LUMO | 0.64 |
|  | B3LYP | 20.5 | 71.2 | 149.9 | 7.61 | 1.7 | 701.8 | 1.11 |  |  |  | 0.75 |

**Table S9**

| **Molecules** | **Methods** | **µ_g_** | **<α>** | **β_0_** | **Δµ_ge_** | **ΔE_ge_** | **λ_max_** | ***f_ge_*** | Orbitals involved in Excitations | | | **C_i_** |
| --- | --- | --- | --- | --- | --- | --- | --- | --- | --- | --- | --- | --- |
| **17** | HF | 29.4 | 42.3 | 378.9 | 18.5 | 2.7 | 465.5 | 1.21 | HOMO | **→** | LUMO | 0.64 |
|  | B3LYP | 19.3 | 55.0 | 106.2 | 31.8 | 2.1 | 574.0 | 1.28 |  |  |  | 0.73 |
| **18** | HF | 30.8 | 46.9 | 652.9 | 19.0 | 2.4 | 508.4 | 1.27 | HOMO | **→** | LUMO | 0.66 |
|  | B3LYP | 19.8 | 57.7 | 121.9 | 32.4 | 2.0 | 600.9 | 1.23 |  |  |  | 0.74 |
| **19** | HF | 37.0 | 27.8 | 88.7 | 39.8 | 2.5 | 487.6 | .076 | HOMO | **→** | LUMO | 0.69 |
|  | B3LYP^*^ | 23.9 | 62.0 | 92.2 | 33.5 | 1.1 | 1120.7 | 0.39 |  |  |  | 0.85 |
| **20** | HF | 30.3 | 35.4 | 97.3 | 31.9 | 2.8 | 444.0 | 0.02 | HOMO | **→** | LUMO | 0.63 |
|  | B3LYP^*^ | 24.8 | 64.1 | 288.9 | 20.7 | 0.3 | 3785.4 | 0.02 |  |  |  | 0.81 |
| **21** | HF | 23.5 | 60.3 | 142.6 | 38.3 | 2.5 | 490.6 | 2.09 | HOMO | **→** | LUMO | 0.66 |
|  | B3LYP | 20.3 | 68.2 | 963.2 | 34.8 | 2.1 | 579.2 | 1.70 |  |  |  | 0.73 |

*For Molecule 19, absorptions were found to be in the near-IR regions, both in the B3LYP and CAM-B3LYP methodologies.

For Molecule 20, in the TD-CAM-B3LYP computation, oscillator strengths for the 1^ST^ and 2^ND^ excited states were found to be zero. Thus, the data shown for this case are from the 3^RD^ excited state.

Also, for the Molecule 20, in B3LYP methodology, the absorption was found to be in extreme far-IR region (3785.4 nm), with a very smaller value of oscillator strength.

**Section S2:** **The optimized geometries of all the molecules were obtained in cartesian coordinates using the ωb97XD approach. Please consult the main text of the article for the visualization of molecular structures and their corresponding numbering for the respective compounds.**

**Molecule 1**

***Atoms***  *X Y Z*

C -2.66563798 0.65457146 0.00088017

C -1.27047800 0.65451618 0.00065112

C -1.27049842 3.07077631 -0.00026497

C -2.66532348 3.07075363 -0.00051498

C -3.36297220 1.86257516 0.00045410

H -3.21543465 -0.29772380 0.00130893

H -0.72100755 -0.29801873 0.00176438

H -0.72026069 4.02289751 -0.00030282

H -3.21540788 4.02305653 -0.00126617

H -4.46257621 1.86280175 0.00045465

C 0.96710773 1.86229045 0.00131304

C 1.66431081 2.85245238 0.69410351

C 1.66493144 0.87241656 -0.69059728

C 3.05902272 2.85305322 0.69437018

H 1.11386728 3.63315528 1.23885728

C 3.06006903 0.87230480 -0.68961367

H 1.11558503 0.09183618 -1.23652935

C 3.75717223 1.86247167 0.00259564

H 3.60863487 3.63384015 1.23989264

H 3.61000475 0.09146866 -1.23490245

N -0.57289214 1.86223954 0.00067800

C 5.86245222 2.82852887 0.67711968

N 6.41334195 3.66550770 1.26157406

C 5.86409096 0.89889138 -0.67038524

N 6.41640175 0.06256568 -1.25443328

C 5.22717188 1.86333353 0.00313292

**Molecule 2**

***Atoms***  *X Y Z*

C 5.27588600 1.05866500 0.54773600

C 3.90377100 1.02011800 0.55408100

C 3.90377200 -1.02012000 -0.55407800

C 5.27588700 -1.05866600 -0.54773100

C 5.98219900 -0.00000100 0.00000400

H 5.77572200 1.89603400 0.99508000

H 3.30638000 1.77793000 1.01658400

H 3.30638200 -1.77793100 -1.01658300

H 5.77572400 -1.89603400 -0.99507600

H 7.05623000 0.00000000 0.00000400

C 1.79475500 -0.00000100 0.00000000

C 1.08375900 -1.15225500 0.34444600

C 1.08376600 1.15225600 -0.34444900

C -0.27992700 -1.15202100 0.34810100

H 1.60656300 -2.04033600 0.65893100

C -0.27992000 1.15202800 -0.34811300

H 1.60657700 2.04033400 -0.65893100

C -1.05508100 0.00000400 -0.00000900

H -0.77486500 -2.04677500 0.66650400

H -0.77485100 2.04678400 -0.66651900

C -2.47103400 0.00000400 -0.00000900

C -3.24179800 1.19951900 -0.14391500

C -3.24179000 -1.19951300 0.14391300

C -4.58974000 1.21726500 -0.14125500

H -2.73564100 2.14481800 -0.23160900

C -4.58973200 -1.21726700 0.14125800

H -2.73562600 -2.14480800 0.23161000

C -5.38075400 -0.00000600 -0.00002000

H -5.13251900 2.14119900 -0.23551300

H -5.13250600 -2.14120100 0.23554300

N 3.22766500 -0.00000400 0.00000500

C -7.31045566 1.16891425 0.13583296

N -7.86205761 2.18255427 0.25360051

C -7.31045207 -1.16893093 -0.13574194

N -7.86205090 -2.18257685 -0.25347329

C -6.67435400 -0.00000494 0.00002463

**Molecule 3a**

***Atoms***  *X Y Z*

C -2.66563798 0.65457146 0.00088017

C -1.27047800 0.65451618 0.00065112

C -1.27049842 3.07077631 -0.00026497

C -2.66532348 3.07075363 -0.00051498

C -3.36297220 1.86257516 0.00045410

H -3.21543465 -0.29772380 0.00130893

H -0.72100755 -0.29801873 0.00176438

H -0.72026069 4.02289751 -0.00030282

H -3.21540788 4.02305653 -0.00126617

H -4.46257621 1.86280175 0.00045465

C 0.96710773 1.86229045 0.00131304

C 1.66493144 0.87241656 -0.69059728

C 3.05902272 2.85305322 0.69437018

C 3.06006903 0.87230480 -0.68961367

H 1.11558503 0.09183618 -1.23652935

C 3.75717223 1.86247167 0.00259564

H 3.60863487 3.63384015 1.23989264

H 3.61000475 0.09146866 -1.23490245

C 5.29717186 1.86337457 0.00315850

C 5.99540103 0.65550676 0.00483325

C 5.99396967 3.07168716 0.00188288

C 7.39011257 0.65604736 0.00591404

H 5.44576561 -0.29693196 0.00661586

C 7.38910743 3.07240000 0.00196784

H 5.44381460 4.02376708 0.00038295

C 8.08723610 1.86486170 0.00412120

H 7.94053302 -0.29596631 0.00786811

H 7.93823442 4.02525452 0.00054644

N -0.57289214 1.86223954 0.00067800

N 1.66431081 2.85245238 0.69410351

C 10.19315591 3.04197598 0.00340541

N 10.74460051 4.06251823 0.00186503

C 10.19351487 0.68841424 0.00787722

N 10.74527076 -0.33195823 0.01021460

C 9.55723570 1.86509722 0.00518177

**Molecule 3b**

***Atoms***  *X Y Z*

C -2.66563798 0.65457146 0.00088017

C -1.27047800 0.65451618 0.00065112

C -1.27049842 3.07077631 -0.00026497

C -2.66532348 3.07075363 -0.00051498

C -3.36297220 1.86257516 0.00045410

H -3.21543465 -0.29772380 0.00130893

H -0.72100755 -0.29801873 0.00176438

H -0.72026069 4.02289751 -0.00030282

H -3.21540788 4.02305653 -0.00126617

H -4.46257621 1.86280175 0.00045465

C 0.96710773 1.86229045 0.00131304

C 1.66431081 2.85245238 0.69410351

C 1.66493144 0.87241656 -0.69059728

H 1.11386728 3.63315528 1.23885728

C 3.06006903 0.87230480 -0.68961367

H 1.11558503 0.09183618 -1.23652935

C 3.75717223 1.86247167 0.00259564

H 3.61000475 0.09146866 -1.23490245

C 5.29717186 1.86337457 0.00315850

C 5.99540103 0.65550676 0.00483325

C 5.99396967 3.07168716 0.00188288

C 7.39011257 0.65604736 0.00591404

H 5.44576561 -0.29693196 0.00661586

C 7.38910743 3.07240000 0.00196784

H 5.44381460 4.02376708 0.00038295

C 8.08723610 1.86486170 0.00412120

H 7.94053302 -0.29596631 0.00786811

H 7.93823442 4.02525452 0.00054644

N -0.57289214 1.86223954 0.00067800

N 3.05902272 2.85305322 0.69437018

C 10.19315591 3.04197598 0.00340541

N 10.74460051 4.06251823 0.00186503

C 10.19351487 0.68841424 0.00787722

N 10.74527076 -0.33195823 0.01021460

C 9.55723570 1.86509722 0.00518177

**Molecule 4**

***Atoms***  *X Y Z*

C -2.66563798 0.65457146 0.00088017

C -1.27047800 0.65451618 0.00065112

C -1.27049842 3.07077631 -0.00026497

C -2.66532348 3.07075363 -0.00051498

C -3.36297220 1.86257516 0.00045410

H -3.21543465 -0.29772380 0.00130893

H -0.72100755 -0.29801873 0.00176438

H -0.72026069 4.02289751 -0.00030282

H -3.21540788 4.02305653 -0.00126617

H -4.46257621 1.86280175 0.00045465

C 0.96710773 1.86229045 0.00131304

C 1.66493144 0.87241656 -0.69059728

C 3.05902272 2.85305322 0.69437018

H 1.11558503 0.09183618 -1.23652935

C 3.75717223 1.86247167 0.00259564

H 3.60863487 3.63384015 1.23989264

C 5.29717186 1.86337457 0.00315850

C 5.99540103 0.65550676 0.00483325

C 5.99396967 3.07168716 0.00188288

C 7.39011257 0.65604736 0.00591404

H 5.44576561 -0.29693196 0.00661586

C 7.38910743 3.07240000 0.00196784

H 5.44381460 4.02376708 0.00038295

C 8.08723610 1.86486170 0.00412120

H 7.94053302 -0.29596631 0.00786811

H 7.93823442 4.02525452 0.00054644

N -0.57289214 1.86223954 0.00067800

N 1.66431081 2.85245238 0.69410351

N 3.06006903 0.87230480 -0.68961367

C 10.19315591 3.04197598 0.00340541

N 10.74460051 4.06251823 0.00186503

C 10.19351487 0.68841424 0.00787722

N 10.74527076 -0.33195823 0.01021460

C 9.55723570 1.86509722 0.00518177

**Molecule 5**

***Atoms***  *X Y Z*

C -2.66563798 0.65457146 0.00088017

C -1.27047800 0.65451618 0.00065112

C -1.27049842 3.07077631 -0.00026497

C -2.66532348 3.07075363 -0.00051498

C -3.36297220 1.86257516 0.00045410

H -3.21543465 -0.29772380 0.00130893

H -0.72100755 -0.29801873 0.00176438

H -0.72026069 4.02289751 -0.00030282

H -3.21540788 4.02305653 -0.00126617

H -4.46257621 1.86280175 0.00045465

C 0.96710773 1.86229045 0.00131304

C 1.66493144 0.87241656 -0.69059728

C 3.06006903 0.87230480 -0.68961367

H 1.11558503 0.09183618 -1.23652935

C 3.75717223 1.86247167 0.00259564

H 3.61000475 0.09146866 -1.23490245

C 5.29717186 1.86337457 0.00315850

C 5.99540103 0.65550676 0.00483325

C 5.99396967 3.07168716 0.00188288

C 7.39011257 0.65604736 0.00591404

H 5.44576561 -0.29693196 0.00661586

C 7.38910743 3.07240000 0.00196784

H 5.44381460 4.02376708 0.00038295

C 8.08723610 1.86486170 0.00412120

H 7.94053302 -0.29596631 0.00786811

H 7.93823442 4.02525452 0.00054644

N -0.57289214 1.86223954 0.00067800

N 1.66431081 2.85245238 0.69410351

N 3.05902272 2.85305322 0.69437018

C 10.19315591 3.04197598 0.00340541

N 10.74460051 4.06251823 0.00186503

C 10.19351487 0.68841424 0.00787722

N 10.74527076 -0.33195823 0.01021460

C 9.55723570 1.86509722 0.00518177

**Molecule 6a**

***Atoms***  *X Y Z*

C -2.66563798 0.65457146 0.00088017

C -1.27047800 0.65451618 0.00065112

C -1.27049842 3.07077631 -0.00026497

C -2.66532348 3.07075363 -0.00051498

C -3.36297220 1.86257516 0.00045410

H -3.21543465 -0.29772380 0.00130893

H -0.72100755 -0.29801873 0.00176438

H -0.72026069 4.02289751 -0.00030282

H -3.21540788 4.02305653 -0.00126617

H -4.46257621 1.86280175 0.00045465

C 0.96710773 1.86229045 0.00131304

C 3.05902272 2.85305322 0.69437018

C 3.06006903 0.87230480 -0.68961367

C 3.75717223 1.86247167 0.00259564

H 3.60863487 3.63384015 1.23989264

H 3.61000475 0.09146866 -1.23490245

C 5.29717186 1.86337457 0.00315850

C 5.99540103 0.65550676 0.00483325

C 5.99396967 3.07168716 0.00188288

C 7.39011257 0.65604736 0.00591404

H 5.44576561 -0.29693196 0.00661586

C 7.38910743 3.07240000 0.00196784

H 5.44381460 4.02376708 0.00038295

C 8.08723610 1.86486170 0.00412120

H 7.94053302 -0.29596631 0.00786811

H 7.93823442 4.02525452 0.00054644

N -0.57289214 1.86223954 0.00067800

N 1.66431081 2.85245238 0.69410351

N 1.66493144 0.87241656 -0.69059728

C 10.19315591 3.04197598 0.00340541

N 10.74460051 4.06251823 0.00186503

C 10.19351487 0.68841424 0.00787722

N 10.74527076 -0.33195823 0.01021460

C 9.55723570 1.86509722 0.00518177

**Molecule 6b**

***Atoms***  *X Y Z*

C -2.66563798 0.65457146 0.00088017

C -1.27047800 0.65451618 0.00065112

C -1.27049842 3.07077631 -0.00026497

C -2.66532348 3.07075363 -0.00051498

C -3.36297220 1.86257516 0.00045410

H -3.21543465 -0.29772380 0.00130893

H -0.72100755 -0.29801873 0.00176438

H -0.72026069 4.02289751 -0.00030282

H -3.21540788 4.02305653 -0.00126617

H -4.46257621 1.86280175 0.00045465

C 0.96710773 1.86229045 0.00131304

C 1.66431081 2.85245238 0.69410351

C 1.66493144 0.87241656 -0.69059728

H 1.11386728 3.63315528 1.23885728

H 1.11558503 0.09183618 -1.23652935

C 3.75717223 1.86247167 0.00259564

C 5.29717186 1.86337457 0.00315850

C 5.99540103 0.65550676 0.00483325

C 5.99396967 3.07168716 0.00188288

C 7.39011257 0.65604736 0.00591404

H 5.44576561 -0.29693196 0.00661586

C 7.38910743 3.07240000 0.00196784

H 5.44381460 4.02376708 0.00038295

C 8.08723610 1.86486170 0.00412120

H 7.94053302 -0.29596631 0.00786811

H 7.93823442 4.02525452 0.00054644

N -0.57289214 1.86223954 0.00067800

N 3.05902272 2.85305322 0.69437018

N 3.06006903 0.87230480 -0.68961367

C 10.19315591 3.04197598 0.00340541

N 10.74460051 4.06251823 0.00186503

C 10.19351487 0.68841424 0.00787722

N 10.74527076 -0.33195823 0.01021460

C 9.55723570 1.86509722 0.00518177

**Molecule 7a**

***Atoms***  *X Y Z*

C -2.66563798 0.65457146 0.00088017

C -1.27047800 0.65451618 0.00065112

C -1.27049842 3.07077631 -0.00026497

C -2.66532348 3.07075363 -0.00051498

C -3.36297220 1.86257516 0.00045410

H -3.21543465 -0.29772380 0.00130893

H -0.72100755 -0.29801873 0.00176438

H -0.72026069 4.02289751 -0.00030282

H -3.21540788 4.02305653 -0.00126617

H -4.46257621 1.86280175 0.00045465

C 0.96710773 1.86229045 0.00131304

C 3.06006903 0.87230480 -0.68961367

C 3.75717223 1.86247167 0.00259564

H 3.61000475 0.09146866 -1.23490245

C 5.29717186 1.86337457 0.00315850

C 5.99540103 0.65550676 0.00483325

C 5.99396967 3.07168716 0.00188288

C 7.39011257 0.65604736 0.00591404

H 5.44576561 -0.29693196 0.00661586

C 7.38910743 3.07240000 0.00196784

H 5.44381460 4.02376708 0.00038295

C 8.08723610 1.86486170 0.00412120

H 7.94053302 -0.29596631 0.00786811

H 7.93823442 4.02525452 0.00054644

N -0.57289214 1.86223954 0.00067800

N 3.05902272 2.85305322 0.69437018

N 1.66431081 2.85245238 0.69410351

N 1.66493144 0.87241656 -0.69059728

C 9.55723570 1.86509722 0.00518177

C 10.22343782 3.09801783 0.00332082

N 10.77488242 4.11856008 0.00178044

C 10.22381388 0.63238172 0.00800557

N 10.77556976 -0.38799076 0.01034296

**Molecule 7b**

***Atoms***  *X Y Z*

C -2.66563798 0.65457146 0.00088017

C -1.27047800 0.65451618 0.00065112

C -1.27049842 3.07077631 -0.00026497

C -2.66532348 3.07075363 -0.00051498

C -3.36297220 1.86257516 0.00045410

H -3.21543465 -0.29772380 0.00130893

H -0.72100755 -0.29801873 0.00176438

H -0.72026069 4.02289751 -0.00030282

H -3.21540788 4.02305653 -0.00126617

H -4.46257621 1.86280175 0.00045465

C 0.96710773 1.86229045 0.00131304

C 1.66431081 2.85245238 0.69410351

H 1.11386728 3.63315528 1.23885728

C 3.75717223 1.86247167 0.00259564

C 5.29717186 1.86337457 0.00315850

C 5.99540103 0.65550676 0.00483325

C 5.99396967 3.07168716 0.00188288

C 7.39011257 0.65604736 0.00591404

H 5.44576561 -0.29693196 0.00661586

C 7.38910743 3.07240000 0.00196784

H 5.44381460 4.02376708 0.00038295

C 8.08723610 1.86486170 0.00412120

H 7.94053302 -0.29596631 0.00786811

H 7.93823442 4.02525452 0.00054644

N -0.57289214 1.86223954 0.00067800

N 3.05902272 2.85305322 0.69437018

N 3.06006903 0.87230480 -0.68961367

N 1.66493144 0.87241656 -0.69059728

C 10.19315591 3.04197598 0.00340541

N 10.74460051 4.06251823 0.00186503

C 10.19351487 0.68841424 0.00787722

N 10.74527076 -0.33195823 0.01021460

C 9.55723570 1.86509722 0.00518177

**Molecule 8**

***Atoms***  *X Y Z*

C 0.49344340 -0.32468791 0.33851051

C 0.58681097 2.08965606 0.36173795

C -1.26663026 0.96242705 -0.70236140

H -2.21600558 1.00469949 -1.25555988

C 1.30340458 3.39249644 0.76261267

C 0.55849815 4.51777589 1.11663526

C 2.69711620 3.44754243 0.77188655

C 1.20723724 5.69799495 1.47918497

H -0.54026657 4.47428576 1.10848102

C 3.34621219 4.62774959 1.13547332

H 3.28437460 2.56068666 0.49306146

C 2.60154777 5.75294078 1.48896545

H 0.62015797 6.58518116 1.75763618

H 4.44510646 4.67073321 1.14309040

C 2.49120924 -1.85097274 0.72037919

C 0.27525325 -2.75694870 1.04700605

C 3.04597284 -3.08482240 1.05960498

H 3.14564873 -1.00747307 0.45682837

C 0.83005546 -3.99096408 1.38731537

H -0.81673385 -2.62784427 1.04198161

C 2.21517611 -4.15509553 1.39353424

H 4.13799260 -3.21431222 1.06426684

H 0.17502255 -4.83427051 1.65045102

H 2.65281808 -5.12783128 1.66102445

N 1.10573354 -1.68700886 0.71372032

N -0.71123265 -0.27128289 -0.36318385

N -0.61733790 2.14296522 -0.34020268

N 1.14216294 0.85540863 0.70149376

C 2.53891434 8.05637180 2.20362314

N 1.89192908 8.97502211 2.49187940

C 4.62259846 7.01427532 1.86960448

N 5.78250082 7.02926121 1.86821310

C 3.28501105 6.99699376 1.87120901

**Molecule 9**

***Atoms***  *X Y Z*

C 0.49344340 -0.32468791 0.33851051

C 0.58681097 2.08965606 0.36173795

H 2.09138567 0.81340090 1.25512548

C 1.30340458 3.39249644 0.76261267

C 0.55849815 4.51777589 1.11663526

C 2.69711620 3.44754243 0.77188655

C 1.20723724 5.69799495 1.47918497

H -0.54026657 4.47428576 1.10848102

C 3.34621219 4.62774959 1.13547332

H 3.28437460 2.56068666 0.49306146

C 2.60154777 5.75294078 1.48896545

H 0.62015797 6.58518116 1.75763618

H 4.44510646 4.67073321 1.14309040

C 2.49120924 -1.85097274 0.72037919

C 0.27525325 -2.75694870 1.04700605

C 3.04597284 -3.08482240 1.05960498

H 3.14564873 -1.00747307 0.45682837

C 0.83005546 -3.99096408 1.38731537

H -0.81673385 -2.62784427 1.04198161

C 2.21517611 -4.15509553 1.39353424

H 4.13799260 -3.21431222 1.06426684

H 0.17502255 -4.83427051 1.65045102

H 2.65281808 -5.12783128 1.66102445

N 1.10573354 -1.68700886 0.71372032

N -0.71123265 -0.27128289 -0.36318385

N -0.61733790 2.14296522 -0.34020268

C 1.14216294 0.85540863 0.70149376

N -1.26663026 0.96242705 -0.70236140

C 2.53891434 8.05637180 2.20362314

N 1.89192908 8.97502211 2.49187940

C 4.62259846 7.01427532 1.86960448

N 5.78250082 7.02926121 1.86821310

C 3.28501105 6.99699376 1.87120901

**Molecule 10**

***Atoms***  *X Y Z*

C -2.66563798 0.65457146 0.00088017

C -1.27047800 0.65451618 0.00065112

C -1.27049842 3.07077631 -0.00026497

C -2.66532348 3.07075363 -0.00051498

C -3.36297220 1.86257516 0.00045410

H -3.21543465 -0.29772380 0.00130893

H -0.72100755 -0.29801873 0.00176438

H -0.72026069 4.02289751 -0.00030282

H -3.21540788 4.02305653 -0.00126617

H -4.46257621 1.86280175 0.00045465

C 0.96710773 1.86229045 0.00131304

C 3.75717223 1.86247167 0.00259564

C 5.29717186 1.86337457 0.00315850

C 5.99540103 0.65550676 0.00483325

C 5.99396967 3.07168716 0.00188288

C 7.39011257 0.65604736 0.00591404

H 5.44576561 -0.29693196 0.00661586

C 7.38910743 3.07240000 0.00196784

H 5.44381460 4.02376708 0.00038295

C 8.08723610 1.86486170 0.00412120

H 7.94053302 -0.29596631 0.00786811

H 7.93823442 4.02525452 0.00054644

N -0.57289214 1.86223954 0.00067800

N 1.66493144 0.87241656 -0.69059728

N 3.06006903 0.87230480 -0.68961367

N 3.05902272 2.85305322 0.69437018

N 1.66431081 2.85245238 0.69410351

C 10.19315591 3.04197598 0.00340541

N 10.74460051 4.06251823 0.00186503

C 10.19351487 0.68841424 0.00787722

N 10.74527076 -0.33195823 0.01021460

C 9.55723570 1.86509722 0.00518177

**Molecule 11**

***Atoms***  *X Y Z*

C 0.08899686 -1.15201055 -0.15610878

C 1.41854681 -1.11307373 0.28625299

C 1.82620737 0.26266646 0.28346608

C 0.73195631 1.01802426 -0.16063750

N -0.32277859 0.14969953 -0.42646608

H -1.21343762 0.41281085 -0.75238447

H 2.02621965 -1.96352802 0.57658648

H 2.79943675 0.64583857 0.57125001

C -2.23752866 -2.15458809 -0.37646018

C -0.33250115 -3.63531421 -0.50397048

C -3.09414262 -3.24099849 -0.55294452

H -2.65042104 -1.14258885 -0.25555032

C -1.18920356 -4.72203063 -0.68150436

H 0.75587865 -3.79082303 -0.48459409

C -2.56984275 -4.52510827 -0.70587673

H -4.18265047 -3.08579689 -0.57190827

H -0.77568478 -5.73393679 -0.80201319

H -3.24544683 -5.38154257 -0.84507358

C 0.59210536 2.53836919 -0.36211456

C -0.67341612 3.12512091 -0.38759458

C 1.73057486 3.32882332 -0.51901453

C -0.80037389 4.50193154 -0.57061352

H -1.57080008 2.50156217 -0.26463803

C 1.60379322 4.70620362 -0.70111289

H 2.72808781 2.86655988 -0.49877992

C 0.33858772 5.29281950 -0.72707192

H -1.79786513 4.96440135 -0.59133598

H 2.50162144 5.32924694 -0.82438384

N -0.85658617 -2.35169754 -0.35161795

C 1.31723014 7.47169188 -1.06658764

N 2.28219076 8.10277431 -1.19381179

C -1.02443365 7.27203114 -0.94002034

N -2.09007033 7.72997576 -0.95749038

C 0.20444755 6.74393416 -0.91987407

**Molecule 12**

***Atoms***  *X Y Z*

C 0.08899686 -1.15201055 -0.15610878

C 1.41854681 -1.11307373 0.28625299

C 1.82620737 0.26266646 0.28346608

C 0.73195631 1.01802426 -0.16063750

H 2.02621965 -1.96352802 0.57658648

H 2.79943675 0.64583857 0.57125001

C -2.23752866 -2.15458809 -0.37646018

C -0.33250115 -3.63531421 -0.50397048

C -3.09414262 -3.24099849 -0.55294452

H -2.65042104 -1.14258885 -0.25555032

C -1.18920356 -4.72203063 -0.68150436

H 0.75587865 -3.79082303 -0.48459409

C -2.56984275 -4.52510827 -0.70587673

H -4.18265047 -3.08579689 -0.57190827

H -0.77568478 -5.73393679 -0.80201319

H -3.24544683 -5.38154257 -0.84507358

C 0.59210536 2.53836919 -0.36211456

C -0.67341612 3.12512091 -0.38759458

C 1.73057486 3.32882332 -0.51901453

C -0.80037389 4.50193154 -0.57061352

H -1.57080008 2.50156217 -0.26463803

C 1.60379322 4.70620362 -0.70111289

H 2.72808781 2.86655988 -0.49877992

C 0.33858772 5.29281950 -0.72707192

H -1.79786513 4.96440135 -0.59133598

H 2.50162144 5.32924694 -0.82438384

N -0.85658617 -2.35169754 -0.35161795

O -0.32277859 0.14969953 -0.42646608

C 1.31723014 7.47169188 -1.06658764

N 2.28219076 8.10277431 -1.19381179

C -1.02443365 7.27203114 -0.94002034

N -2.09007033 7.72997576 -0.95749038

C 0.20444755 6.74393416 -0.91987407

**Molecule 13**

***Atoms***  *X Y Z*

C 0.08899686 -1.15201055 -0.15610878

C 1.41854681 -1.11307373 0.28625299

C 1.82620737 0.26266646 0.28346608

C 0.73195631 1.01802426 -0.16063750

H 2.02621965 -1.96352802 0.57658648

H 2.79943675 0.64583857 0.57125001

C -2.23752866 -2.15458809 -0.37646018

C -0.33250115 -3.63531421 -0.50397048

C -3.09414262 -3.24099849 -0.55294452

H -2.65042104 -1.14258885 -0.25555032

C -1.18920356 -4.72203063 -0.68150436

H 0.75587865 -3.79082303 -0.48459409

C -2.56984275 -4.52510827 -0.70587673

H -4.18265047 -3.08579689 -0.57190827

H -0.77568478 -5.73393679 -0.80201319

H -3.24544683 -5.38154257 -0.84507358

C 0.59210536 2.53836919 -0.36211456

C -0.67341612 3.12512091 -0.38759458

C 1.73057486 3.32882332 -0.51901453

C -0.80037389 4.50193154 -0.57061352

H -1.57080008 2.50156217 -0.26463803

C 1.60379322 4.70620362 -0.70111289

H 2.72808781 2.86655988 -0.49877992

C 0.33858772 5.29281950 -0.72707192

H -1.79786513 4.96440135 -0.59133598

H 2.50162144 5.32924694 -0.82438384

N -0.85658617 -2.35169754 -0.35161795

S -0.32277859 0.14969953 -0.42646608

C 1.31723014 7.47169188 -1.06658764

N 2.28219076 8.10277431 -1.19381179

C -1.02443365 7.27203114 -0.94002034

N -2.09007033 7.72997576 -0.95749038

C 0.20444755 6.74393416 -0.91987407

**Molecule 14a**

***Atoms***  *X Y Z*

C 0.08899686 -1.15201055 -0.15610878

C 1.41854681 -1.11307373 0.28625299

C 0.73195631 1.01802426 -0.16063750

N -0.32277859 0.14969953 -0.42646608

H -1.21343762 0.41281085 -0.75238447

H 2.02621965 -1.96352802 0.57658648

C -2.23752866 -2.15458809 -0.37646018

C -0.33250115 -3.63531421 -0.50397048

C -3.09414262 -3.24099849 -0.55294452

H -2.65042104 -1.14258885 -0.25555032

C -1.18920356 -4.72203063 -0.68150436

H 0.75587865 -3.79082303 -0.48459409

C -2.56984275 -4.52510827 -0.70587673

H -4.18265047 -3.08579689 -0.57190827

H -0.77568478 -5.73393679 -0.80201319

H -3.24544683 -5.38154257 -0.84507358

C 0.59210536 2.53836919 -0.36211456

C -0.67341612 3.12512091 -0.38759458

C 1.73057486 3.32882332 -0.51901453

C -0.80037389 4.50193154 -0.57061352

H -1.57080008 2.50156217 -0.26463803

C 1.60379322 4.70620362 -0.70111289

H 2.72808781 2.86655988 -0.49877992

C 0.33858772 5.29281950 -0.72707192

H -1.79786513 4.96440135 -0.59133598

H 2.50162144 5.32924694 -0.82438384

N -0.85658617 -2.35169754 -0.35161795

N 1.82620737 0.26266646 0.28346608

C 1.31723014 7.47169188 -1.06658764

N 2.28219076 8.10277431 -1.19381179

C -1.02443365 7.27203114 -0.94002034

N -2.09007033 7.72997576 -0.95749038

C 0.20444755 6.74393416 -0.91987407

**Molecule 14b**

***Atoms***  *X Y Z*

C 0.08899686 -1.15201055 -0.15610878

C 1.82620737 0.26266646 0.28346608

C 0.73195631 1.01802426 -0.16063750

N -0.32277859 0.14969953 -0.42646608

H -1.21343762 0.41281085 -0.75238447

H 2.79943675 0.64583857 0.57125001

C -2.23752866 -2.15458809 -0.37646018

C -0.33250115 -3.63531421 -0.50397048

C -3.09414262 -3.24099849 -0.55294452

H -2.65042104 -1.14258885 -0.25555032

C -1.18920356 -4.72203063 -0.68150436

H 0.75587865 -3.79082303 -0.48459409

C -2.56984275 -4.52510827 -0.70587673

H -4.18265047 -3.08579689 -0.57190827

H -0.77568478 -5.73393679 -0.80201319

H -3.24544683 -5.38154257 -0.84507358

C 0.59210536 2.53836919 -0.36211456

C -0.67341612 3.12512091 -0.38759458

C 1.73057486 3.32882332 -0.51901453

C -0.80037389 4.50193154 -0.57061352

H -1.57080008 2.50156217 -0.26463803

C 1.60379322 4.70620362 -0.70111289

H 2.72808781 2.86655988 -0.49877992

C 0.33858772 5.29281950 -0.72707192

H -1.79786513 4.96440135 -0.59133598

H 2.50162144 5.32924694 -0.82438384

N -0.85658617 -2.35169754 -0.35161795

N 1.41854681 -1.11307373 0.28625299

C 1.31723014 7.47169188 -1.06658764

N 2.28219076 8.10277431 -1.19381179

C -1.02443365 7.27203114 -0.94002034

N -2.09007033 7.72997576 -0.95749038

C 0.20444755 6.74393416 -0.91987407

**Molecule 15a**

***Atoms***  *X Y Z*

C 0.08899686 -1.15201055 -0.15610878

C 1.41854681 -1.11307373 0.28625299

C 0.73195631 1.01802426 -0.16063750

H 2.02621965 -1.96352802 0.57658648

C -2.23752866 -2.15458809 -0.37646018

C -0.33250115 -3.63531421 -0.50397048

C -3.09414262 -3.24099849 -0.55294452

H -2.65042104 -1.14258885 -0.25555032

C -1.18920356 -4.72203063 -0.68150436

H 0.75587865 -3.79082303 -0.48459409

C -2.56984275 -4.52510827 -0.70587673

H -4.18265047 -3.08579689 -0.57190827

H -0.77568478 -5.73393679 -0.80201319

H -3.24544683 -5.38154257 -0.84507358

C 0.59210536 2.53836919 -0.36211456

C -0.67341612 3.12512091 -0.38759458

C 1.73057486 3.32882332 -0.51901453

C -0.80037389 4.50193154 -0.57061352

H -1.57080008 2.50156217 -0.26463803

C 1.60379322 4.70620362 -0.70111289

H 2.72808781 2.86655988 -0.49877992

C 0.33858772 5.29281950 -0.72707192

H -1.79786513 4.96440135 -0.59133598

H 2.50162144 5.32924694 -0.82438384

N -0.85658617 -2.35169754 -0.35161795

O -0.32277859 0.14969953 -0.42646608

N 1.82620737 0.26266646 0.28346608

C 1.31723014 7.47169188 -1.06658764

N 2.28219076 8.10277431 -1.19381179

C -1.02443365 7.27203114 -0.94002034

N -2.09007033 7.72997576 -0.95749038

C 0.20444755 6.74393416 -0.91987407

**Molecule 15b**

***Atoms***  *X Y Z*

C 0.08899686 -1.15201055 -0.15610878

C 1.82620737 0.26266646 0.28346608

C 0.73195631 1.01802426 -0.16063750

H 2.79943675 0.64583857 0.57125001

C -2.23752866 -2.15458809 -0.37646018

C -0.33250115 -3.63531421 -0.50397048

C -3.09414262 -3.24099849 -0.55294452

H -2.65042104 -1.14258885 -0.25555032

C -1.18920356 -4.72203063 -0.68150436

H 0.75587865 -3.79082303 -0.48459409

C -2.56984275 -4.52510827 -0.70587673

H -4.18265047 -3.08579689 -0.57190827

H -0.77568478 -5.73393679 -0.80201319

H -3.24544683 -5.38154257 -0.84507358

C 0.59210536 2.53836919 -0.36211456

C -0.67341612 3.12512091 -0.38759458

C 1.73057486 3.32882332 -0.51901453

C -0.80037389 4.50193154 -0.57061352

H -1.57080008 2.50156217 -0.26463803

C 1.60379322 4.70620362 -0.70111289

H 2.72808781 2.86655988 -0.49877992

C 0.33858772 5.29281950 -0.72707192

H -1.79786513 4.96440135 -0.59133598

H 2.50162144 5.32924694 -0.82438384

N -0.85658617 -2.35169754 -0.35161795

O -0.32277859 0.14969953 -0.42646608

N 1.41854681 -1.11307373 0.28625299

C 1.31723014 7.47169188 -1.06658764

N 2.28219076 8.10277431 -1.19381179

C -1.02443365 7.27203114 -0.94002034

N -2.09007033 7.72997576 -0.95749038

C 0.20444755 6.74393416 -0.91987407

**Molecule 16a**

***Atoms***  *X Y Z*

C 0.08899686 -1.15201055 -0.15610878

C 1.41854681 -1.11307373 0.28625299

C 0.73195631 1.01802426 -0.16063750

H 2.02621965 -1.96352802 0.57658648

C -2.23752866 -2.15458809 -0.37646018

C -0.33250115 -3.63531421 -0.50397048

C -3.09414262 -3.24099849 -0.55294452

H -2.65042104 -1.14258885 -0.25555032

C -1.18920356 -4.72203063 -0.68150436

H 0.75587865 -3.79082303 -0.48459409

C -2.56984275 -4.52510827 -0.70587673

H -4.18265047 -3.08579689 -0.57190827

H -0.77568478 -5.73393679 -0.80201319

H -3.24544683 -5.38154257 -0.84507358

C 0.59210536 2.53836919 -0.36211456

C -0.67341612 3.12512091 -0.38759458

C 1.73057486 3.32882332 -0.51901453

C -0.80037389 4.50193154 -0.57061352

H -1.57080008 2.50156217 -0.26463803

C 1.60379322 4.70620362 -0.70111289

H 2.72808781 2.86655988 -0.49877992

C 0.33858772 5.29281950 -0.72707192

H -1.79786513 4.96440135 -0.59133598

H 2.50162144 5.32924694 -0.82438384

N -0.85658617 -2.35169754 -0.35161795

S -0.32277859 0.14969953 -0.42646608

N 1.82620737 0.26266646 0.28346608

C 1.31723014 7.47169188 -1.06658764

N 2.28219076 8.10277431 -1.19381179

C -1.02443365 7.27203114 -0.94002034

N -2.09007033 7.72997576 -0.95749038

C 0.20444755 6.74393416 -0.91987407

**Molecule 16b**

***Atoms***  *X Y Z*

C 0.08899686 -1.15201055 -0.15610878

C 1.82620737 0.26266646 0.28346608

C 0.73195631 1.01802426 -0.16063750

H 2.79943675 0.64583857 0.57125001

C -2.23752866 -2.15458809 -0.37646018

C -0.33250115 -3.63531421 -0.50397048

C -3.09414262 -3.24099849 -0.55294452

H -2.65042104 -1.14258885 -0.25555032

C -1.18920356 -4.72203063 -0.68150436

H 0.75587865 -3.79082303 -0.48459409

C -2.56984275 -4.52510827 -0.70587673

H -4.18265047 -3.08579689 -0.57190827

H -0.77568478 -5.73393679 -0.80201319

H -3.24544683 -5.38154257 -0.84507358

C 0.59210536 2.53836919 -0.36211456

C -0.67341612 3.12512091 -0.38759458

C 1.73057486 3.32882332 -0.51901453

C -0.80037389 4.50193154 -0.57061352

H -1.57080008 2.50156217 -0.26463803

C 1.60379322 4.70620362 -0.70111289

H 2.72808781 2.86655988 -0.49877992

C 0.33858772 5.29281950 -0.72707192

H -1.79786513 4.96440135 -0.59133598

H 2.50162144 5.32924694 -0.82438384

N -0.85658617 -2.35169754 -0.35161795

S -0.32277859 0.14969953 -0.42646608

N 1.41854681 -1.11307373 0.28625299

C 1.31723014 7.47169188 -1.06658764

N 2.28219076 8.10277431 -1.19381179

C -1.02443365 7.27203114 -0.94002034

N -2.09007033 7.72997576 -0.95749038

C 0.20444755 6.74393416 -0.91987407

**Molecule 17**

***Atoms***  *X Y Z*

C -0.68185289 0.76507435 -0.11499003

C 0.64402774 0.76504963 -0.12467377

H -1.27543879 -0.15891852 -0.10634143

H 1.23761366 1.68904252 -0.13331838

C -1.51418159 2.06077066 -0.11499013

C -2.89075684 2.00552617 -0.33512659

C -0.89190667 3.28952768 0.10518761

C -3.64470356 3.17889129 -0.33573845

H -3.38092748 1.03671859 -0.50944359

C -1.64609300 4.46324515 0.10558642

H 0.19301209 3.33323330 0.27888310

C -3.02226851 4.40813843 -0.11496111

H -4.72964201 3.13548867 -0.50986852

H -1.15524841 5.43188181 0.27962331

C 2.85293152 -0.47539802 -0.34483554

C 0.85408112 -1.75940824 0.09545226

C 3.60687806 -1.64876324 -0.34547134

H 3.34310232 0.49341305 -0.51913269

C 1.60826727 -2.93312583 0.09582712

H -0.23083766 -1.80311725 0.26914678

C 2.98444281 -2.87801480 -0.12471919

H 4.69181653 -1.60535723 -0.51960045

H 1.11742253 -3.90176598 0.26984417

H 3.57911827 -3.80303221 -0.12520796

N 1.47635625 -0.53064681 -0.12470031

C -3.81720253 5.64465754 -0.11558925

C -3.15810963 6.86071807 0.10961131

N -2.61254964 7.86730449 0.29601966

C -5.19703193 5.54968705 -0.34136212

N -6.33917701 5.47107583 -0.52824418

**Molecule 18**

***Atoms***  *X Y Z*

C -1.51418159 2.06077066 -0.11499013

C -2.46685072 2.27783470 -1.11086972

C -1.31574199 3.01726463 0.88064491

C -3.22039170 3.45146046 -1.11142196

H -2.62251786 1.52390525 -1.89610849

C -2.07012123 4.19085818 0.88079929

H -0.56505676 2.84626555 1.66573090

C -3.02224729 4.40815206 -0.11508039

H -3.97087431 3.62290535 -1.89671108

H -1.91373048 4.94464861 1.66617573

C -3.81667907 5.64499380 -0.11589315

C -3.59035588 6.58305202 0.90034824

N -3.40301829 7.35952377 1.74153563

C -4.76327976 5.82832048 -1.13287910

N -5.54682251 5.98006798 -1.97468280

C -0.68185289 0.76507435 -0.11499003

C -0.03263650 -0.24556878 -0.11498995

C 1.75228420 -1.75837866 0.88095272

C 0.60117549 -2.49780859 -1.11056193

C 2.50661670 -2.93149596 0.88085865

H 1.90909016 -1.00371766 1.66526162

C 1.35496514 -3.67178088 -1.11023491

H -0.14969848 -2.32693075 -1.89549383

C 2.30767850 -3.88869749 -0.11483476

H 3.25791791 -3.10241501 1.66547938

H 1.19793754 -4.42598042 -1.89509134

H 2.90252055 -4.81360789 -0.11466944

N 0.79969220 -1.54126509 -0.11498985

**Molecule 19**

***Atoms***  *X Y Z*

C -1.51418159 2.06077066 -0.11499013

C -2.89075684 2.00552617 -0.33512659

C -0.89190667 3.28952768 0.10518761

C -3.64470356 3.17889129 -0.33573845

H -3.38092748 1.03671859 -0.50944359

C -1.64609300 4.46324515 0.10558642

H 0.19301209 3.33323330 0.27888310

C -3.02226851 4.40813843 -0.11496111

H -4.72964201 3.13548867 -0.50986852

H -1.15524841 5.43188181 0.27962331

C -3.81720253 5.64465754 -0.11558925

C -3.15810963 6.86071807 0.10961131

N -2.61254964 7.86730449 0.29601966

C -5.19703193 5.54968705 -0.34136212

N -6.33917701 5.47107583 -0.52824418

C -0.68185289 0.76507435 -0.11499003

H -1.28718547 -0.04769467 0.22834506

H 0.16013409 0.88203311 0.53483962

C -0.19012988 0.47082515 -1.54440456

H 0.41077279 1.28602089 -1.88976218

H -1.03218696 0.34853326 -2.19316073

C 2.02743943 -0.75672419 -1.33959629

C 0.03004413 -2.05439216 -1.74506931

C 2.78741917 -1.92619074 -1.33887454

H 2.51496488 0.21612935 -1.18111379

C 0.78997209 -3.22439925 -1.74339459

H -1.05665240 -2.10500362 -1.90525094

C 2.16847777 -3.16048532 -1.54048053

H 3.87427031 -1.87579603 -1.17915135

H 0.30187740 -4.19701715 -1.90230407

H 2.76789264 -4.08243851 -1.53981128

N 0.64867489 -0.82068740 -1.54303791

**Molecule 20**

***Atoms***  *X Y Z*

C -1.51418159 2.06077066 -0.11499013

C -2.46685072 2.27783470 -1.11086972

C -1.31574199 3.01726463 0.88064491

C -3.22039170 3.45146046 -1.11142196

H -2.62251786 1.52390525 -1.89610849

C -2.07012123 4.19085818 0.88079929

H -0.56505676 2.84626555 1.66573090

C -3.02224729 4.40815206 -0.11508039

H -3.97087431 3.62290535 -1.89671108

H -1.91373048 4.94464861 1.66617573

C -3.81667907 5.64499380 -0.11589315

C -3.59035588 6.58305202 0.90034824

N -3.40301829 7.35952377 1.74153563

C -4.76327976 5.82832048 -1.13287910

N -5.54682251 5.98006798 -1.97468280

C -0.68185289 0.76507435 -0.11499003

H -0.83569222 0.03255923 -0.87961743

C 0.24477160 0.54943849 0.85011891

C 1.17139610 0.33380264 1.81522785

H 2.17884631 0.66750835 1.67890263

C 1.72511827 -0.61723949 4.10768245

C -0.53958142 -0.82998525 3.29293522

C 1.36473442 -1.27687581 5.28251229

H 2.76005783 -0.27409326 3.96487999

C -0.90047036 -1.48896498 4.46848394

H -1.29013970 -0.65398096 2.50883460

C 0.05144120 -1.71258327 5.46316794

H 2.11526862 -1.45340960 6.06662373

H -1.93558358 -1.83222505 4.61056616

H -0.23248792 -2.23260886 6.38958792

N 0.77303613 -0.39402290 3.11260186

**Molecule 21**

***Atoms***  *X Y Z*

C -2.08101061 1.29001485 0.25702493

C -3.03129686 1.51031906 -0.74041857

C -2.39830170 1.54512425 1.59111401

C -4.29834102 1.98626760 -0.40382711

H -2.78058669 1.30982891 -1.79217420

C -3.66601157 2.02028444 1.92807747

H -1.64950634 1.37137639 2.37740035

C -4.61596474 2.24100956 0.93087820

H -5.04721270 2.16053382 -1.19003255

H -3.91603346 2.22094761 2.98007442

C -5.95151837 2.74257122 1.28532482

C -6.23475036 2.98709966 2.63584600

N -6.46919383 3.18950652 3.75373137

C -6.87917258 2.95278648 0.25615565

N -7.64703250 3.12679084 -0.59573263

C -0.68185289 0.76507435 -0.11499003

H -0.44050007 0.56992876 -1.13898564

C 0.24477160 0.54943849 0.85011891

C 1.17139609 0.33380263 1.81522785

C 2.09802058 0.11816677 2.78033679

H 1.85666777 0.31331236 3.80433241

C 4.44746455 -0.62707794 3.40576533

C 3.81439702 -0.66209217 1.07425551

C 5.71488016 -1.10195356 3.06905693

H 4.19731733 -0.42496177 4.45734377

C 5.08183021 -1.13805152 0.73737914

H 3.06548456 -0.48868253 0.28800603

C 6.03209517 -1.35787605 1.73448028

H 6.46416453 -1.27502779 3.85513248

H 5.33152471 -1.33966034 -0.31451476

H 7.03145285 -1.73235022 1.46924515

N 3.49717831 -0.40677373 2.40832182
